# PoseBusters: AI-based docking methods fail to generate physically valid poses or generalise to novel sequences†

**DOI:** 10.1039/d3sc04185a

**Published:** 2023-12-13

**Authors:** Martin Buttenschoen, Garrett M. Morris, Charlotte M. Deane

**Affiliations:** a Department of Statistics 24-29 St Giles' Oxford OX1 3LB UK deane@stats.ox.ac.uk

## Abstract

The last few years have seen the development of numerous deep learning-based protein–ligand docking methods. They offer huge promise in terms of speed and accuracy. However, despite claims of state-of-the-art performance in terms of crystallographic root-mean-square deviation (RMSD), upon closer inspection, it has become apparent that they often produce physically implausible molecular structures. It is therefore not sufficient to evaluate these methods solely by RMSD to a native binding mode. It is vital, particularly for deep learning-based methods, that they are also evaluated on steric and energetic criteria. We present PoseBusters, a Python package that performs a series of standard quality checks using the well-established cheminformatics toolkit RDKit. The PoseBusters test suite validates chemical and geometric consistency of a ligand including its stereochemistry, and the physical plausibility of intra- and intermolecular measurements such as the planarity of aromatic rings, standard bond lengths, and protein–ligand clashes. Only methods that both pass these checks and predict native-like binding modes should be classed as having “state-of-the-art” performance. We use PoseBusters to compare five deep learning-based docking methods (DeepDock, DiffDock, EquiBind, TankBind, and Uni-Mol) and two well-established standard docking methods (AutoDock Vina and CCDC Gold) with and without an additional post-prediction energy minimisation step using a molecular mechanics force field. We show that both in terms of physical plausibility and the ability to generalise to examples that are distinct from the training data, no deep learning-based method yet outperforms classical docking tools. In addition, we find that molecular mechanics force fields contain docking-relevant physics missing from deep-learning methods. PoseBusters allows practitioners to assess docking and molecular generation methods and may inspire new inductive biases still required to improve deep learning-based methods, which will help drive the development of more accurate and more realistic predictions.

## Abstract 2

PoseBusters assesses molecular poses using steric and energetic criteria. We find that classical protein-ligand docking tools currently still outperform deep learning-based methods.

## Graphical abstract

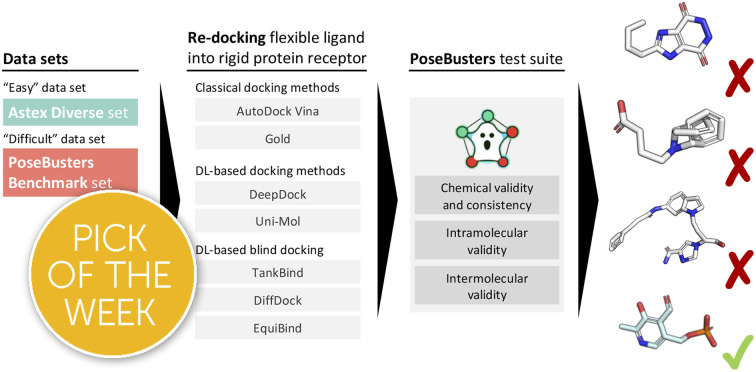

## 1 Introduction

Docking, an essential step in structure-based drug discovery,^1^ is the task of predicting the predominant binding modes of a protein–ligand complex given an experimentally solved or computationally modelled protein structure and a ligand structure.^2^ The predicted complexes are often used in a virtual screening workflow to help select molecules from a large library of possible candidates;^3^ or directly by medicinal chemists to understand the binding mode and to decide whether a small molecule is a suitable drug candidate.^4^

Docking methods are designed with the understanding that binding is enabled by interactions between target and ligand structures but due to the complexity of this property methods tend to strike a balance between fast calculation and accuracy.^5^ Deep learning (DL) promises to disrupt the dominant design principle of classical docking software, and DL-based docking methods promise to unlock fast and accurate virtual screening for drug discovery. To this end, a handful of different DL-based docking methods have already been proposed.^6–10^

Classical non-DL-based docking methods include within their search and scoring functions terms that help ensure chemical consistency and physical plausibility; for example limiting the degrees of movement in the ligand to only the rotatable bonds in the ligand and including penalties if the protein and ligand clash.^11,12^ Some current DL-based docking methods, as we will show, still lack such key “inductive biases” resulting in the creation of unrealistic poses despite obtaining root-mean-squared deviation (RMSD) values from the experimental binding mode that are less than the widely-used 2 Å threshold.^13^ To assess such docking methods, an independent test suite is necessary to check the chemical consistency and physical plausibility alongside established metrics, such as the binding mode RMSD. Such a test suite would help the field to identify missing inductive biases required to improve DL-based docking methods, driving the development of more accurate and realistic docking predictions.

The problem of assessing the physical plausibility of docking predictions is akin to the structure validation of ligand data in the Protein Data Bank (PDB).^14,15^ Structure validation assesses the agreement of the ligands bond lengths and angles with those observed in related chemical structures and the presence of steric clashes both within the ligand and between it and its surroundings.^15^ While these tests were designed for users to select those ligand crystal structures which are likely to be correct,^15^ docking methods are evaluated on their ability to recover crystal structures so their output should pass the same physical plausibility tests.

Physical plausibility checks are also part of some workflows for conformation generation.^16,17^ Friedrich *et al.* use geometry checks performed by NAOMI^18^ which measures—like the PDB tests mention above—the deviation from known optimal values for bond lengths and bond angles, and also tests for divergences from the planarity of aromatic rings.^17^

In addition to physical checks, chemical checks are also needed.^19^ Chemical checks proposed for checking PDB structures include the identification of mislabelled stereo assignment, inconsistent bonding patterns, missing functional groups, and unlikely ionisation states.^19^ The problem of checking chemical plausibility has also come up in *de novo* molecule generation, where Brown *et al.* proposed a test suite including checks for the chemical validity of any proposed molecule.^20^ For docking, the focus is less on stability and synthetic accessibility of a molecular structure as it is hoped that these have been tested prior to attempting docking, but more on chemical consistency and physical realism of the predicted bound conformation.

Some comparisons of docking methods have included additional metrics based on volume overlap^21^ or protein–ligand interactions^22^ to supplement pose accuracy-based metrics such as RMSD of atomic positions and run time measurements, but the majority of comparisons of docking methods are predominantly based on binding mode RMSD.^13,23–25^

The current standard practice of comparing docking methods based on RMSD-based metrics alone also extends to the introduction papers of recent new methods. The five DL-based docking methods we test in this paper^6–10^ all claim better performance than standard docking methods but these claims rest entirely on RMSD. None of these methods test their outputs for physical plausibility.

In this paper we present PoseBusters, a test suite that is designed to identify implausible conformations and ligand poses. We used PoseBusters to evaluate the predicted ligand poses generated by the five DL-based docking methods (DeepDock,^6^ DiffDock,^7^ EquiBind,^8^ TankBind,^9^ and Uni-Mol^10^) and two standard non-DL-based docking methods (AutoDock Vina^12^ and Gold^26^). These poses were generated by re-docking the cognate ligands of the 85 protein–ligand crystal complexes in the Astex Diverse set^27^ and 308 ligands of the protein–ligand crystal complexes in the PoseBusters Benchmark set, a new set of complexes released from 2021 onwards, into their cognate receptor crystal structures. On the commonly-used Astex Diverse set, the DL-based docking method DiffDock appears to perform best in terms of RMSD alone but when taking physical plausibility into account, Gold and AutoDock Vina perform best. On the PoseBusters Benchmark set, a test set that is harder because it contains only complexes that the DL methods have not been trained on, Gold and AutoDock Vina are the best methods in terms of RMSD alone and when taking physical plausibility into account or when proteins with novel sequences are considered. The DL-based methods make few valid predictions for the unseen complexes. Overall, we show that no DL-based method yet outperforms standard docking methods when consideration of physical plausibility is taken into account. The PoseBusters test suite will enable DL method developers to better understand the limitations of current methods, ultimately resulting in more accurate and realistic predictions.

## 2 Methods

Five DL-based and two classical docking methods were used to re-dock known ligands into their respective proteins and the predicted ligand poses were evaluated with the PoseBusters test suite. The following section describes the docking methods, the data sets, and the PoseBusters test suite for checking physicochemical consistency and structural plausibility of the generated poses.

### 2.1 Docking methods

The selected five DL-based docking methods^6–10^ cover a wide range of DL-based approaches for pose prediction. Table 1 lists the methods and their publications. In order to examine the ability of standard non-DL-based methods to predict accurate chemically and physically valid poses, we also included the well-established docking methods AutoDock Vina^28^ and Gold.^29^

**Table 1:** Selected DL-based docking methods. The selection includes five methodologically different DL-based docking methods published over the last two years

Method	Authors	Date	Search space
DeepDock^6^	Méndez-Lucio *et al.*	Dec 2021	Pocket
DiffDock^7^	Corso *et al.*	Feb 2023	Blind
EquiBind^8^	Stärk *et al.*	Feb 2022	Blind
TankBind^9^	Lu *et al.*	Oct 2022	Blind
Uni-Mol^10^	Zhou *et al.*	Feb 2023	Pocket

The five DL-based docking methods can be summarised as follows. Full details of each can be found in their respective references. DeepDock^6^ learns a statistical potential based on the distance likelihood between ligand heavy atoms and points of the mesh of the surface of the binding pocket. DiffDock^7^ uses equivariant graph neural networks in a diffusion process for blind docking. EquiBind^8^ applies equivariant graph neural networks for blind docking. TankBind^9^ is a blind docking method that uses a trigonometry-aware neural network for docking in each pocket predicted by a binding pocket prediction method. Uni-Mol^10^ carries out docking with SE3-equivariant transformers. All five DL-based docking methods are trained on subsets of the PDBbind General Set^30^ as detailed in Table 2. DeepDock is trained on v2019 and the other four are trained on v2020. It should be noted that we used the DL models as trained by the respective authors without further hyperparameter tuning.

**Table 2:** Data sets used to train the selected five machine learning-based docking methods. All five DL-based methods were trained on subsets of the PDBBind 2020 General Set

Method	Training and validation set
DeepDock	PDBBind 2019 General Set without complexes included in CASF-2016 or those that fail pre-processing—16 367 complexes
DiffDock, EquiBind	PDBbind 2020 General Set keeping complexes published before 2019 and without those with ligands found in test set—17 347 complexes
TankBind	PDBbind 2020 General Set keeping complexes published before 2019 and without those failing pre-processing—18 755 complexes
Uni-Mol	PDBBind 2020 General Set without complexes where protein sequence identity (MMSeq2) with CASF-2016 is above 40% and ligand fingerprint similarity is above 80%—18 404 complexes

The docking protocols that were used to generate predictions with each method and the software versions used are given in section S1 of the ESI.†Table 3 lists the search space definitions that we used for each method. DeepDock and Uni-Mol require the definition of a binding site while DiffDock, EquiBind, and TankBind are ‘blind’ docking methods that search over the entire protein. We used the default search spaces for DiffDock, DeepDock, EquiBind, and TankBind but larger than default search spaces for AutoDock Vina, Gold, and Uni-Mol such that they are more comparable with the blind docking methods. ESI Fig. S1† shows the search spaces for one example protein–ligand complex. We show results for Uni-Mol across a range of binding site definitions starting from their preferred definition of all residues with an atom within 6 Å of a heavy atom of the crystal ligand. Under this tight pocket definition Uni-Mol performs better than any of the blind docking methods (ESI Fig. S21†).

**Table 3:** Search spaces of the docking methods used

Method	Search space
**Classical docking methods**	
Gold	Sphere of radius 25 Å centered on the geometric centre of the crystal ligand heavy atoms
Vina	Cube with side length 25 Å centered on the geometric centre of crystal ligand heavy atoms

**DL-based docking methods**	
DeepDock	Protein surface mesh nodes within 10 Å of any crystal ligand atom
Uni-Mol	Protein residues within 8 Å of any crystal ligand heavy atom

**DL-based blind docking methods**	
DiffDock	Entire crystal protein
EquiBind	Chains of crystal protein which are within 10 Å of any crystal ligand heavy atom
TankBind	Pockets identified by P2Rank^31^

### 2.2 The PoseBusters test suite

The PoseBusters test suite is organised into three groups of tests. The first checks chemical validity and contains tests for the chemical validity and consistency relative to the input. The second group checks intramolecular properties and tests for the ligand geometry and the ligand conformation's energy computed using the universal force field (UFF).^32^ The third group considers intermolecular interactions and checks for protein–ligand and ligand–cofactor clashes. Descriptions of all the tests PoseBusters performs in the three sections are listed in Table 4. Molecule poses which pass all tests in PoseBusters are ‘PB-valid’.

**Table 4:** Description of the checks used in the PoseBusters test suite

Test name	Description
**Chemical validity and consistency**	
File loads	The input molecule can be loaded into a molecule object by RDKit
Sanitisation	The input molecule passes RDKit's chemical sanitisation checks
Molecular formula	The molecular formula of the input molecule is the same as that of the true molecule
Bonds	The bonds in the input molecule are the same as in the true molecule
Tetrahedral chirality	The specified tetrahedral chirality in the input molecule is the same as in the true molecule
Double bond stereochemistry	The specified double bond stereochemistry in the input molecule is the same as in the true molecule

**Intramolecular validity**	
Bond lengths	The bond lengths in the input molecule are within 0.75 of the lower and 1.25 of the upper bounds determined by distance geometry
Bond angles	The angles in the input molecule are within 0.75 of the lower and 1.25 of the upper bounds determined by distance geometry
Planar aromatic rings	All atoms in aromatic rings with 5 or 6 members are within 0.25 Å of the closest shared plane
Planar double bonds	The two carbons of aliphatic carbon–carbon double bonds and their four neighbours are within 0.25 Å of the closest shared plane
Internal steric clash	The interatomic distance between pairs of non-covalently bound atoms is above 0.7 of the lower bound determined by distance geometry
Energy ratio	The calculated energy of the input molecule is no more than 100 times the average energy of an ensemble of 50 conformations generated for the input molecule. The energy is calculated using the UFF^32^ in RDKit and the conformations are generated with ETKDGv3 followed by force field relaxation using the UFF with up to 200 iterations

**Intermolecular validity**	
Minimum protein–ligand distance	The distance between protein–ligand atom pairs is larger than 0.75 times the sum of the pairs van der Waals radii
Minimum distance to organic cofactors	The distance between ligand and organic cofactor atoms is larger than 0.75 times the sum of the pairs van der Waals radii
Minimum distance to inorganic cofactors	The distance between ligand and inorganic cofactor atoms is larger than 0.75 times the sum of the pairs covalent radii
Volume overlap with protein	The share of ligand volume that intersects with the protein is less than 7.5%. The volumes are defined by the van der Waals radii around the heavy atoms scaled by 0.8
Volume overlap with organic cofactors	The share of ligand volume that intersects with organic cofactors is less than 7.5%. The volumes are defined by the van der Waals radii around the heavy atoms scaled by 0.8
Volume overlap with inorganic cofactors	The share of ligand volume that intersects with inorganic cofactors is less than 7.5%. The volumes are defined by the van der Waals radii around the heavy atoms scaled by 0.5

For evaluating docking predictions, PoseBusters requires three input files: an SDF file containing the re-docked ligands, an SDF file containing the true ligand(s), and a PDB file containing the protein with any cofactors. The three files are loaded into RDKit molecule objects with the sanitisation option turned off.

#### 2.2.1 Chemical validity and consistency

The first test in PoseBusters checks whether the ligand passes the RDKit's sanitisation. The RDKit's sanitisation processes information on the valency, aromaticity, radicals, conjugation, hybridization, chirality tags, and protonation to check whether a molecule can be represented as an octet-complete Lewis dot structure.^33^ Passing the RDKit's sanitisation is a commonly-used test for chemical validity in cheminformatics, for example in *de novo* molecular generation.^20^

The next test in PoseBusters checks for docking-relevant chemical consistency between the predicted and the true ligands by generating ‘standard InChI’ strings^34^ for the input and output ligands after removing isotopic information and neutralising charges by adding or removing hydrogens where possible. InChI is the *de facto* standard for molecular comparison,^35^ and the ‘standard InChI’ strings generated include the layers for the molecular formula (/), molecular bonds (/c), hydrogens (/h), net charge (/q), protons (/p), tetrahedral chirality (/t), and double bond stereochemistry (/b). Standardisation of the ligand's protonation and charge state is needed because the stereochemistry layer is dependent on the hydrogen (/h), net charge (/q) and proton (/p) layers. These can unexpectedly change during docking even though most docking software considers the charge distribution and protonation state of a ligand as fixed.^12,36^ The normalisation protocol also removes the stereochemistry information of double bonds in primary ketimines which only depends on the hydrogen atom's ambiguous location.

#### 2.2.2 Intramolecular validity

The first set of physical plausibility tests in the PoseBusters test suite validates bond lengths, bond angles, and internal distances between non-covalently bound pairs of atoms in the docked ligand against the corresponding limits in the distance bounds matrix obtained from the RDKit's Distance Geometry module. To pass the tests, all molecular measurements must lie within the user-specified tolerances. The tolerance used throughout this manuscript is 25% for bond lengths and bond angles and 30% for non-covalently bound pairs of atoms *e.g.*: if a bond is less than 75% of the distance geometry bond length lower bound, it is treated as anomalous. This was selected as all but one of the crystal ligands in the Astex Diverse set and all of those in the PoseBusters Benchmark set pass at this threshold.

The PoseBusters test for flatness checks that groups of atoms lie in a plane by calculating the closest plane to the atoms and checking that all atoms are within a user-defined distance from this plane. This test is performed for 5- and 6-membered aromatic rings and non-ring non-aromatic carbon–carbon double bonds. The chosen threshold of 0.25 Å admits all Astex Diverse and PoseBusters Benchmark set crystal structures by a wide margin and as with all other thresholds can be adjusted by the user.

The final test for intramolecular physicochemical plausibility carried out by PoseBusters is an energy calculation to detect unlikely conformations. Our metric for this is the ratio of the energy of the docked ligand conformation to the mean of the energies of a set of 50 generated unconstrained conformations as in Wills *et al.*^37^ The conformations are generated using the RDKit's ETKDGv3 conformation generator^38^ followed by a force field relaxation using the UFF^32^ and up to 200 iterations. The test suite rejects conformations for which this ratio is larger than a user-specified threshold. Wills *et al.* set a ratio of 7 based on the value where 95% of the crystal ligands in the PDBbind data set are considered plausible.^37^ We selected a less strict ratio of 100 where only one structure each from the Astex Diverse and PoseBusters Benchmark set is rejected.

#### 2.2.3 Intermolecular validity

Intermolecular interactions are evaluated by two sets of tests in the PoseBusters test suite. The first set checks the minimum distance between molecules and the second checks the share of overlapping volume. Both sets of tests report on intermolecular interactions of the ligand with three types of molecules: the protein, organic cofactors, and inorganic cofactors.

For the distance-based intermolecular tests PoseBusters calculates the ratio of the pairwise distance between pairs of heavy atoms of two molecules and the sum of the two atoms' van der Waals radii. If this ratio is smaller than a user-defined threshold then the test fails. The default threshold is 0.75 for all pairings. For inorganic cofactor–ligand pairings the covalent radii are used. All crystal structures in the Astex Diverse set and all but one in the PoseBusters Benchmark set pass at this threshold.

For the second set of intermolecular checks, PoseBusters calculates the share of the van der Waals volume of the heavy atoms of the ligand that overlaps with the van der Waals volume of the heavy atoms of the protein using the RDKit's ShapeTverskyIndex function. The tests have a configurable scaling factor for the volume-defining van der Waals radii and a threshold that defines how much overlap constitutes a clash. A threshold is necessary because many crystal structures already contain clashes. For example, Verdonk *et al.* found that 81 out of 305 selected high-quality protein–ligand complexes from the PDB contain steric clashes.^26^ The overlap threshold is 7.5% for all molecule pairings and the scaling factor is 0.8 for protein–ligand and organic cofactor–ligand pairings and 0.5 for inorganic cofactor–ligand pairings.

### 2.3 Quality of fit

PoseBusters calculates the minimum heavy-atom symmetry-aware root-mean-square deviation (RMSD) between the predicted ligand binding mode and the closest crystallographic ligand using the RDKit's GetBestRMS function. Coverage, a metric often used for testing docking methods, is the share of predictions that are within a user adjustable threshold which by default is 2 Å RMSD. This value is arbitrary but commonly-used and recommended for regular-size ligands.^13^

### 2.4 Sequence identity

In this paper, sequence identity between two amino acid chains is the number of exact residue matches after sequence alignment divided by the number of residues of the query sequence. The sequence alignment used is the Smith–Waterman algorithm^39^ implemented in Biopython^40^ using an open gap score of −11 and an extension gap score of −1 and the BLOSUM62 substitution matrix. Unknown amino acid residues are counted as mismatches.

### 2.5 Molecular mechanics energy minimisation

Post-docking energy minimisation of the ligand structure in the binding pocket was performed using the AMBER ff14sb force field^41^ and the Sage small molecule force field^42^ in OpenMM.^43^ The protein files were prepared using PDBfixer^43^ and all protein atom positions were fixed in space only allowing updates to the ligand atoms positions. Minimisation was performed until energy convergence within 0.01 kJ mol^−1^.

### 2.6 Data

#### 2.6.1 Astex Diverse set

The Astex Diverse set^27^ published in 2007 is a set of hand-picked, relevant, diverse, and high-quality protein–ligand complexes from the PDB.^14^ The complexes were downloaded from the PDB as MMTF files^44^ and PyMOL^45^ was used to remove solvents and all occurrences of the ligand of interest from the complexes before saving the proteins with the cofactors in PDB files and the ligands in SDF files.

#### 2.6.2 PoseBusters Benchmark set

The PoseBusters Benchmark set is a new set of carefully-selected publicly-available crystal complexes from the PDB. It is a diverse set of recent high-quality protein–ligand complexes which contain drug-like molecules. It only contains complexes released since 2021 and therefore does not contain any complexes present in the PDBbind General Set v2020 used to train many of the methods. Table S2† lists the steps used to select the 308 unique proteins and 308 unique ligands in the PoseBusters Benchmark set. The complexes were downloaded from the PDB as MMTF files and PyMOL was used to remove solvents and all occurrences of the ligand of interest before saving the proteins with the cofactors in PDB files and the ligands in SDF files.

## 3 Results

The following section presents the analysis of the PoseBusters test suite on the re-docked ligands of five DL-based docking methods and two standard non-DL-based docking methods on the 85 ligands of the Astex Diverse set and the 308 ligands of the PoseBusters Benchmark set into the receptors crystal structures.

### 3.1 Results on the Astex Diverse set

**Fig. 1:**
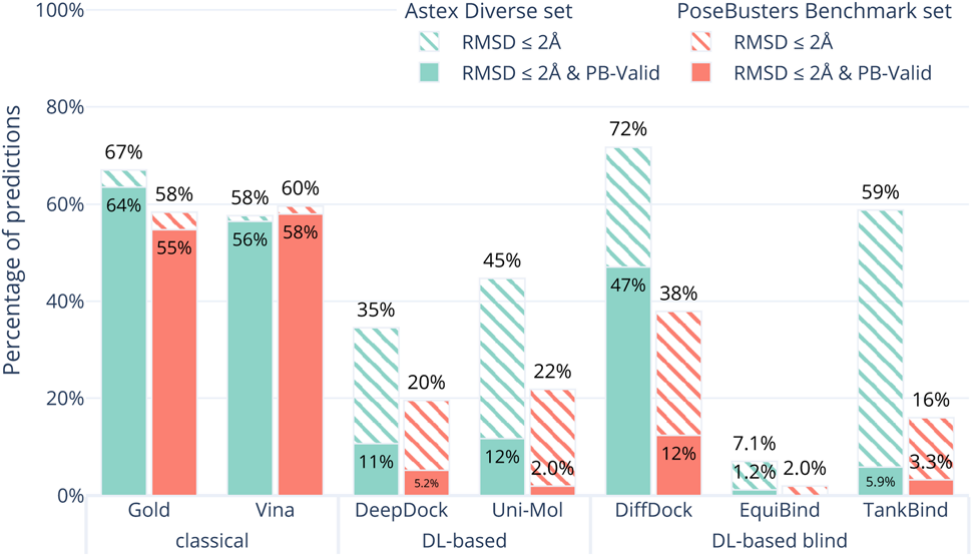
Comparative performance of the docking methods. The Astex Diverse set (85 cases) was chosen as an easy test set containing many complexes the five DL-based methods were trained on while the PoseBusters Benchmark set (308 cases) was chosen to be a difficult test set containing complexes none of the methods was trained on. The striped bars show the share of predictions of each method that have an RMSD within 2 Å and the solid bars show the subset that in addition have valid geometries and energies, *i.e.*, pass all PoseBuster tests and are therefore ‘PB-Valid’. DiffDock appears to outperform the classical methods on the Astex Diverse set when only binding mode RMSD is considered (striped teal bars). However, when physical plausibility is also considered (solid teal bars) *or* when presented with the PoseBusters Benchmark set (coral bars), AutoDock Vina and Gold outperform all DL-based methods.

Fig. 1 shows the overall results of the seven (AutoDock Vina,^12^ Gold,^26^ DeepDock,^6^ DiffDock,^7^ EquiBind,^8^ TankBind,^9^ Uni-Mol^10^) docking methods on the Astex Diverse set in ocean green. The striped bars show the performance only in terms of RMSD coverage (RMSD ≤ 2 Å) and the solid bars show the performance after also considering physical plausibility, *i.e.*, only predictions which in addition pass all tests in PoseBusters and are therefore PB-valid.

The Astex Diverse set is a well-established and commonly-used benchmark for evaluating docking methods. Good performance on this set is expected because the five DL-based methods evaluated here have been trained on most of these complexes. 47 of the 85 complexes in the Astex Diverse set are in the PDBbind 2020 General Set and 67 out of the 85 of the Astex Diverse set proteins have more than 95% sequence identity with proteins found in PDBbind 2020 General Set. AutoDock Vina may also perform well on this data set because the linear regression model behind the scoring function was trained on an earlier version of PDBbind^12^ which already included most of the Astex Diverse set.

The RMSD criterion alone (striped green bars in Fig. 1) gives the impression that DiffDock (72%) performs better than TankBind (59%), Gold (67%), AutoDock Vina (58%) and Uni-Mol (45%). However, when we look closer, accepting only ligand binding modes that are physically sensible, *i.e.*, those predictions that pass all PoseBusters tests and are therefore PB-valid (solid green bars in Fig. 1), many of the apparently impressive DL predictions are removed. The best three methods when considering RMSD and physical plausibility are Gold (64%), AutoDock Vina (56%), and DiffDock (47%) followed by Uni-Mol (12%), DeepDock (11%) and TankBind (5.9%). DiffDock is therefore the only DL-based method that has comparable performance to the standard methods on the Astex Diverse set when considering physical plausibility of the predicted poses.

**Fig. 2:**
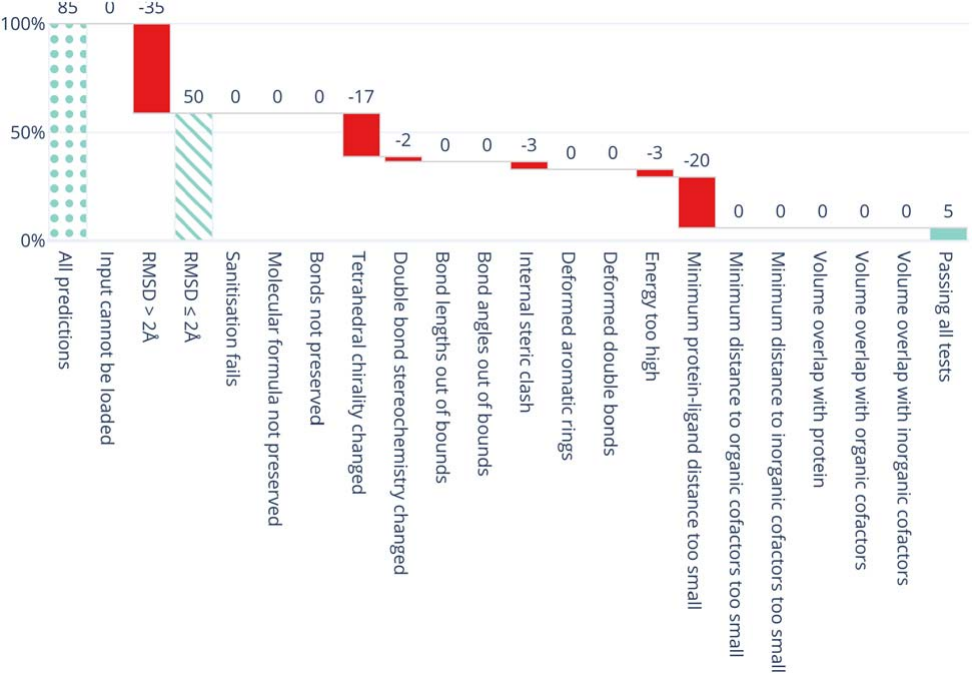
Waterfall plot showing the PoseBusters tests as filters for the TankBind predictions on the Astex Diverse data set. The tests in the PoseBuster test suits are described in Table 4. The leftmost (dotted) bar shows the number of complexes in the test set. The red bars show the number of predictions that fail with each additional test going from left to right. The rightmost (solid) bar indicates the number of predictions that pass all tests, *i.e.* those that are ‘PB-Valid’. For the 85 test cases in the Astex Diverse set 50 (59%) predictions have RMSD within 2 Å RMSD and 5 (5.9%) pass all tests. Fig. S5 and S6 in the ESI† show waterfall plots for all methods and both data sets.

**Fig. 3:**
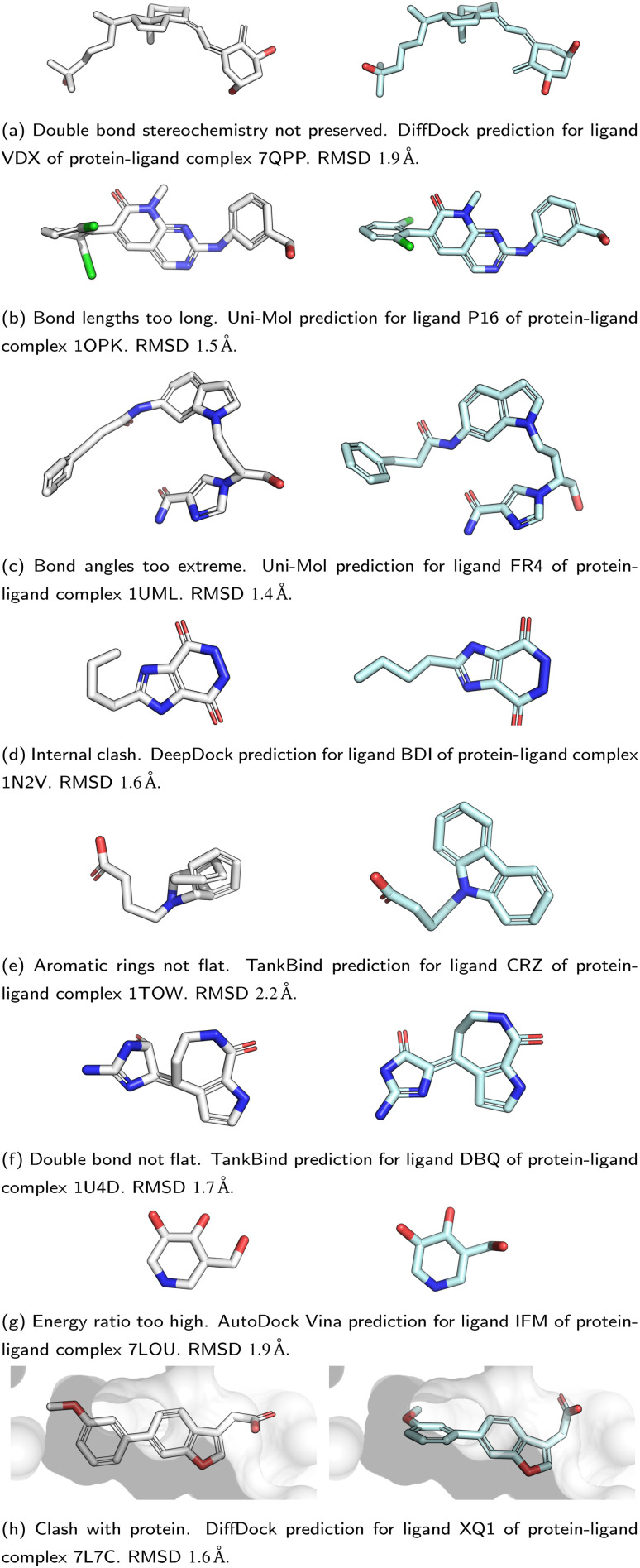
Examples of failure modes that PoseBusters is able to detect. Predictions are shown on the left with white carbons and the crystal structures on the right have cyan carbons. Oxygen atoms are red, nitrogen atoms are dark blue, chlorine atoms are green. Most of the shown predictions have a RMSD within 2 Å but all are physically invalid.

All five DL-based docking methods struggle with physical plausibility, but even the poses produced by the classical methods Gold and AutoDock Vina do not always pass all the checks. Fig. 2 shows a waterfall plot that indicates how many predicted binding modes fail each test. The waterfall plots for the remaining methods are shown in ESI Fig. S5.† The DL-based methods fail on different tests. TankBind habitually overlooks stereochemistry, Uni-Mol very often fails to predict valid bond lengths, and EquiBind tends to produce protein–ligand clashes. The classical methods Gold and AutoDock Vina pass most tests but also generated a few protein–ligand clashes. Fig. 3 shows examples of poses generated by the methods illustrating various failure modes.

The results on the Astex Diverse set suggest that despite what the RMSD ≤ 2 Å criterion would indicate, no DL-based method outperforms classical docking methods when the physical plausibility of the ligand binding mode is taken into account. However, DiffDock in particular is capable of making a large number of useful predictions.

### 3.2 Results on the PoseBusters Benchmark set

The results of the seven (AutoDock Vina, Gold, DeepDock, DiffDock, EquiBind, TankBind, Uni-Mol) docking methods on the PoseBusters Benchmark set are shown in coral in Fig. 1. The striped bars show the performance only in terms of coverage (RMSD within 2 Å) and the solid bars show the performance in terms of PB-validity (passing all of PoseBusters tests).

The PoseBusters Benchmark set was designed to contain no complexes that are in the training data for any of the methods. Performing well on this data set requires a method to be able to generalise well.

All methods perform worse on the PoseBusters Benchmark set than the Astex Diverse set. Gold (55%) and AutoDock Vina (58%) perform the best out of the seven methods with (solid coral bars) and without (striped coral bars) considering PB-validity. On the PoseBusters Benchmark set, the best performing DL method, DiffDock (12%), does not compete with the two standard docking methods. Gold and AutoDock Vina again pass the most tests but for a few protein–ligand clashes.

The waterfall plots in ESI Fig. S6† show which tests fail for each method on the PoseBusters Benchmark set. Again, the methods have different merits and shortcomings. Out of the five DL-based methods, DiffDock still produces the most physically valid poses but few predictions lie within the 2 Å RMSD threshold. EquiBind, Uni-Mol and TankBind generate almost no physically valid poses that pass all tests. Uni-Mol has a relatively good RMSD score (22%) but struggles to predict planar aromatic rings and correct bond lengths.

**Fig. 4:**
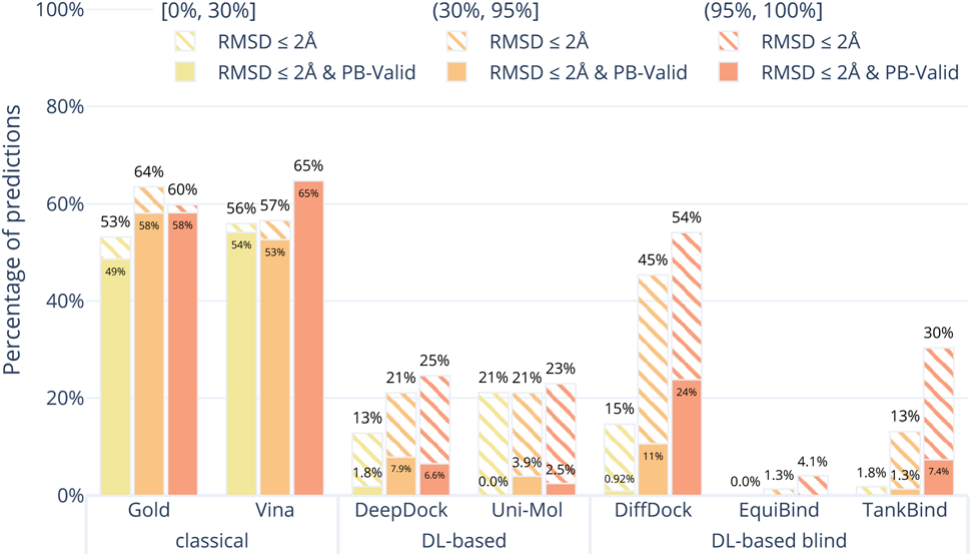
Comparative performance of docking methods on the PoseBusters Benchmark set stratified by sequence identity relative to the PDBBind General Set v2020. The sequence identity is the maximum sequence identity between all chains in the PoseBuster test protein and all chains in the PDBBind General Set v2020. The striped bars show the share of predictions of each method that have an RMSD within 2 Å and the solid bars show those predictions which in addition pass all PoseBuster tests and are therefore PB-valid. The DL-based methods perform far better on proteins that are similar to those they were trained on.

Fig. 4 shows the results of the docking methods on the PoseBusters Benchmark set but stratified by the target protein receptor's maximum sequence identity with the proteins in the PDBbind 2020 General Set.^30^ As the DL-based methods were all trained on subsets of the PDBbind 2020 General Set, this roughly quantifies how different the test set protein targets are from those that the methods were trained on. We bin the test cases into three categories low [0, 30%], medium [30%, 90%], and high [90%, 100%] maximum percentage sequence identity. Without considering physical plausibility (striped bars), the classical methods appear to perform as well on the three protein similarity bins while the DL-based methods perform worse on the proteins with lower sequence identity. This suggests that the DL-based methods are overfitting to the protein targets in their training sets.

We also compared the performance of the docking methods on the PoseBusters Benchmark set stratified by whether protein–ligand complexes contain cofactors (ESI Fig. S3†). Here, we loosely define cofactors as non-protein non-ligand compounds such as metal ions, iron–sulfur clusters, and organic small molecules in the crystal complex within 4.0 Å of any ligand heavy atom. About 45% of protein–ligand complexes in the PoseBusters Benchmark set have a cofactor (ESI Fig. S2†). The classical methods perform slightly better when a cofactor is present while the DL-based docking methods perform worse on those systems.

### 3.3 Results with pose-docking energy minimisation

**Fig. 5:**
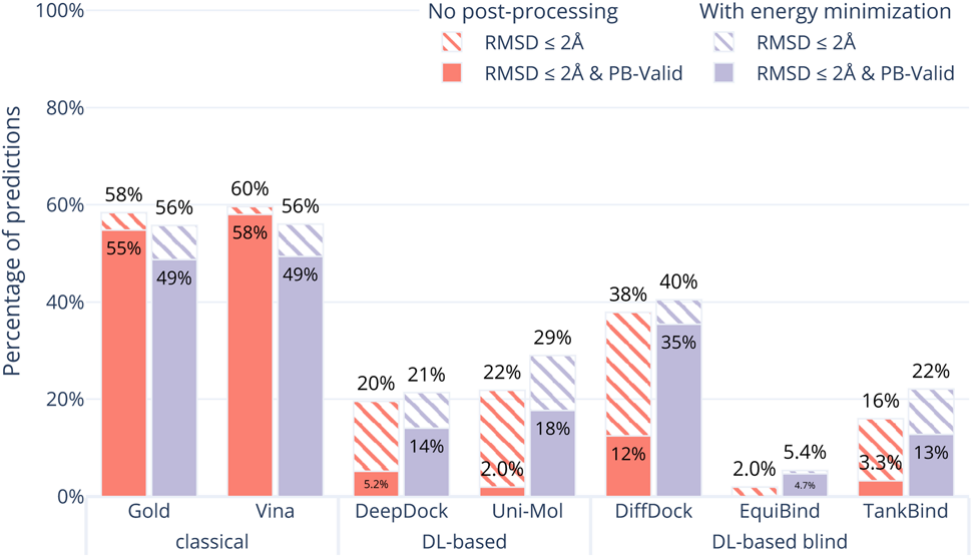
Comparative performance of docking methods with post-docking energy minimisation of the ligand (while keeping the protein fixed) on the PoseBusters Benchmark set. The striped bars show the share of predictions of each method that have an RMSD within 2 Å of the crystal pose and the solid bars show those predictions which in addition pass all PoseBuster tests and are therefore PB-valid. Post-docking energy minimisation significantly improves the relative physical plausibility of the DL-based methods' predictions. This indicates that force fields contain docking-relevant physics which is missing from DL-based methods.

**Fig. 6:**
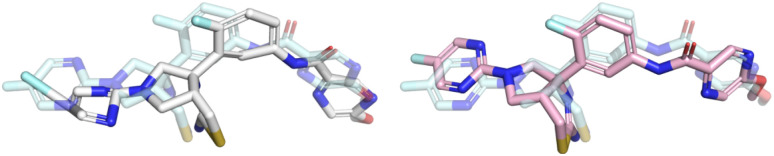
Example of a prediction that was fixed by the post-docking energy minimisation. The Uni-Mol prediction (RMSD 2.0 Å) is shown in white, the optimised prediction (RMSD 1.1 Å) is shown in pink, and the crystal ligand is shown for reference in light blue. Note how the aromatic rings are flattened and the leftmost bond is shortened by the optimisation making the prediction pass all PoseBusters checks.

In order to examine whether the outputs of the DL-based methods can be made physically plausible we performed an additional post-docking energy minimisation of the ligand structures in the binding pocket for the PoseBusters Benchmark set (Fig. 5). Again, striped bars indicate predictions with RMSD ≤ 2 Å while solid bars indicate which of those are also PB-valid *i.e.*, pass all PoseBusters tests. The figure shows that post-docking energy minimisation significantly increases the number of physically plausible structures of the DL-based methods DiffDock, DeepDock, TankBind, and Uni-Mol but does not improve the poses predicted by AutoDock Vina and Gold. We also performed energy minimisation on the Uni-Mol results for the minimal 6 Å pocket (ESI Fig. S22†). The number of poses that pass the tests increase to about the same level as DiffDock. The fact that energy minimisation is able to repair many of the DL methods predicted poses and increase coverage shows that at least some force field physics is missing from DL-based docking methods. An example of a predicted pose that was fixed is shown in Fig. 6. However, even with the energy minimisation step, the best DL-based docking method DiffDock still performs worse than the classical methods Gold and AutoDock Vina.

## 4 Discussion

We present PoseBusters, a test suite designed and built to identify chemically inconsistent and physically implausible ligand poses predicted by protein–ligand docking and molecular generation methods. We show the results of applying the PoseBusters test suite to the output of seven different docking methods, five current DL-based docking methods (DeepDock, DiffDock, EquiBind, TankBind, and Uni-Mol) and two standard methods (AutoDock Vina and Gold).

We find that no DL-based docking method yet outperforms standard docking methods when *both* physical plausibility and binding mode RMSD is taken into account. Our work demonstrates the need for physical plausibility to be taken into account when assessing docking tools because it is possible to perform well on an RMSD-based metric while predicting physically implausible ligand poses (Fig. 3). Using the tests in the PoseBusters test suite as an additional criterion when developing DL-based docking methods will help improve methods and the development of more accurate and realistic predictions.

In addition, the individual tests in the PoseBusters test suite highlight docking-relevant failure modes. The results show that Uni-Mol for example predicts non-standard bond lengths and TankBind creates internal ligand clashes. The ability to identify such failure modes in predicted ligand poses makes PoseBusters a helpful tool for developers to identify inductive biases that could improve their binding mode prediction methods.

Our results also show that, unlike classical docking methods, DL-based docking methods do not generalise well to novel data. The performance of the DL-based methods on the PoseBusters Benchmark set overall was poor and the subset of the PoseBusters Benchmark set with low sequence identity to PDBbind 2020 revealed that DL-based methods are prone to overfitting to the proteins they were trained on. Our analysis of the targets with sequence identity lower than 30% to any member of PDBbind General Set v2020 revealed that across all of the DL-based docking methods almost no physically valid poses were generated within the 2 Å threshold.

The most commonly-used train–test approach for building DL-based docking models is time-based, *e.g.*, complexes released before a certain date are used for training and complexes released later for testing. Based on our results, we argue that this is insufficient for testing generalisation to novel targets and the sequence identity between the proteins in the training and test must be reported on.

Post-docking energy minimisation of the ligand using force fields can considerably improve the docking poses generated by DL-based methods. However, even with an energy minimisation step, the best DL-based method, DiffDock, does not outperform classical docking methods like Gold and AutoDock Vina. This shows that at least some key aspects of chemistry and physics encoded in force fields are missing from deep learning models.

The PoseBusters test suite provides a new criterion, PB-validity, beyond the traditional “RMSD ≤ 2 Å” rule to evaluate the predictions of new DL-based methods, and hopefully will help to identify inductive biases needed for the field to improve docking and molecular generation methods, ultimately resulting in more accurate and realistic predictions. The next generation of DL-based docking methods should aim to outperform standard docking tools on both RMSD criteria and in terms of chemical consistency, physical plausibility, and generalisability.

## Data availability

PoseBusters is made available as a pip-installable Python package and as open source code under the BSD-3-Clause license at https://github.com/maabuu/posebusters. Data for this paper, including the Astex Diverse set and PoseBusters Benchmark set, as well as the individual tabulated test results for each docking are available at Zenodo at https://zenodo.org/records/8278563.

## Author contributions

The manuscript was conceptualised and written through contributions of all authors. M. B. performed the computational experiments, created the PoseBusters software, and wrote the original draft. C. M. D. and G. M. M. supervised the work and reviewed and edited the manuscript.

## Conflicts of interest

There are no conflicts to declare.

## Supplementary Material

SC-015-D3SC04185A-s001
