# Supplementary material for: PoseBusters: AI-based docking methods fail to generate physically valid poses or generalise to novel sequences
Source: Chem Sci. 2023 Dec 13;15(9):3130–9. doi: 10.1039/d3sc04185a (PMC10901501; doi:10.1039/d3sc04185a)
Supplement: SC-015-D3SC04185A-s001 [file SC-015-D3SC04185A-s001.pdf]

Electronic Supplementary Material (ESI) for Chemical Science.
This journal is © The Royal Society of Chemistry 2024

This version of the ESI published on 24/01/2024 replaces the previous version published on 13/12/2023 to include Section S9.

PoseBusters

Supplementary information

Martin Buttenschoen, Garrett M. Morris, and Charlotte M. Deane

Department of Statistics, 24-29 St Giles', Oxford OX1 3LB, United Kingdom

Contents

S1 Docking protocols 1

S2 Search space illustrations 4

S3 PoseBusters Benchmark set procurement 5

S4 Data sets description 6

S5 Data sets 7

S6 Energy minimisation example 8

S7 Cofactor analysis 9

S8 Detailed results 10

S9 Alternative binding site definitions for Uni-Mol 25

S1 Docking protocols

The following protocols detail how the seven docking methods were used to re-dock the ligands into the crystal structures of the Astex Diverse set and the PoseBusters Benchmark set. Methods that require an initial ligand conformation were given identical starting conformations generated with the RDKit's ETKDGv3 conformer generator[1] followed by an energy minimisation using the universal force field (UFF)[2]. All docking protocols were given receptors prepared without waters as none of the DL-based methods supports docking with waters.

AutoDock Vina

Software version Vina 1.2.3, Meeko 0.4.0, Reduce 4.9.210817, ADFRsuite 1.0, RDKit 2022.09.1

Ligand preparation The initial ligand conformations described above were prepared with Meeko using standard settings.

Protein preparation Hydrogen atoms were added with reduce and then the PDBQT files were generated with the ADFR `prepare_receptor` script.

Parameters A bounding box with side-length 25 Å was created around the centroid of the crystal ligand. Vina was used to create 40 poses with an exhaustiveness setting of 32 and the top-ranked pose was selected.

CCDC Gold

Software version CCDC Python API version 3.0.14

Ligand preparation The initial ligand conformations described above were prepared with `LigandPreparation` using the default settings which include adding missing hydrogens, removing unknown atoms, and rule-based protonation of the ligand.

Protein preparation The protein and co-factors were loaded from separate files and all hydrogens were added.

Parameters A settings file was created for each complex using the `Docker` class default settings. The binding site was defined around the crystal ligand centroid using `BindingSiteFromPoint` with radius 25 Å. The settings used are rescore function `plp`, autoscale 100 %, and early termination off. After generating 40 poses only the top-ranked pose was saved.

DeepDock

Software version DeepDock commit hash `54a2a64` from authors' public code repository `https://github.com/OptiMaL-PSE-Lab/DeepDock`, MSMS 2.6.1, PDB2PQR 2.1.1, APBS 3.4.1

Ligand preparation The generated starting ligand conformations were used without further processing.

Protein preparation The steps in example notebook `Docking_example.ipynb` were used to generate protein surface meshes. The function `compute_inp_surface` generated binding site surfaces using the crystal ligands and the crystal protein structures with a distance threshold of 10 Å.

Parameters The protocol and settings in notebook `Docking_example.ipynb` in the DeepDock repository were used for docking.

DiffDock

Software version DiffDock commit hash `fff8f0b` from authors' public code repository `https://github.com/gcorso/DiffDock`

Ligand preparation The generated starting ligand conformations were used without further processing.

Protein preparation ESM was used to generate FASTA files.

Parameters The protocol in `README.md` was used to generate ESM embeddings and then to do inference. 40 poses were sampled using 20 inference steps with no noise on the final step. The top-ranked pose was selected.

EquiBind

Software version EquiBind commit hash `41bd00f` from authors' public code repository `https://github.com/HannesStark/EquiBind`, Reduce 3.3.160602, Open Babel 3.1.0, RDKit 2022.09.1

Ligand preparation The generated starting ligand conformations were processed with Open Babel and then with the RDKit to add missing hydrogens.

Protein preparation The receptors were processed with Open Babel. Then reduce was used to correct receptor residues and to add hydrogens. Then the protein chains which have at least one residue within 10 Å of the crystal ligand were selected.

Parameters The configuration file `configs_clean/inference.yml` in the repository was used.

TankBind

Software version TANKBind commit hash `804e9fc` from authors' public code repository `https://github.com/luwei0917/TankBind`, p2rank 2.3

Ligand preparation The notebook `prediction_example_using_PDB_6hd6.ipynb` was used to renumber the ligand atoms and generate features from the ligands.

Protein preparation The notebook `prediction_example_using_PDB_6hd6.ipynb` was used to generate features from the crystal protein structures.

Parameters The steps in the notebook `prediction_example_using_PDB_6hd6.ipynb` were used for inference. The steps are running p2rank to generate a list of binding pockets and then docking using the TankBind model.

Uni-Mol

Software version Uni-Mol commit hash `b962451` from authors' public code repository `https://github.com/dptech-corp/Uni-Mol`

Ligand preparation The ligands were generated according to the protocol described in the `README.md` file in the top folder of the Uni-Mol repository.

Protein preparation The binding pockets residues are those within 8 Å of any crystal ligand heavy atom.

Parameters The default arguments (`recycling=3`, `batch_size=8`, `dist_threshold=8.0`) were used.

S2 Search space illustrations

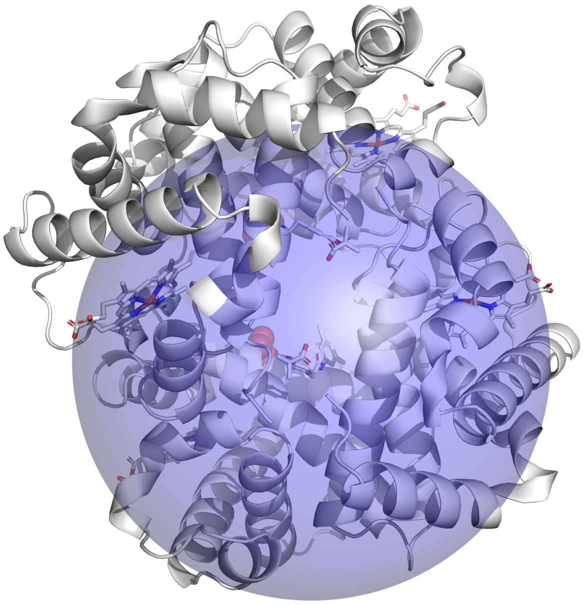

(a) Gold: Sphere of radius 25 Å centered on the geometric centre of the crystal ligand heavy atoms.

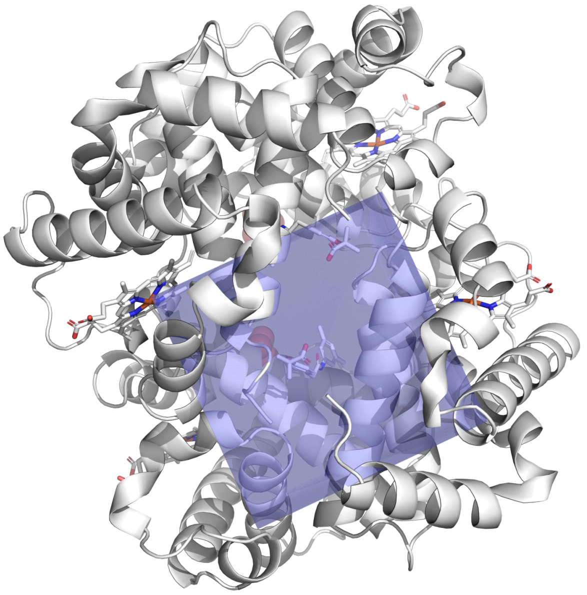

(b) AutoDock Vina: Cube with side length 25 Å centered on the geometric centre of crystal ligand heavy atoms.

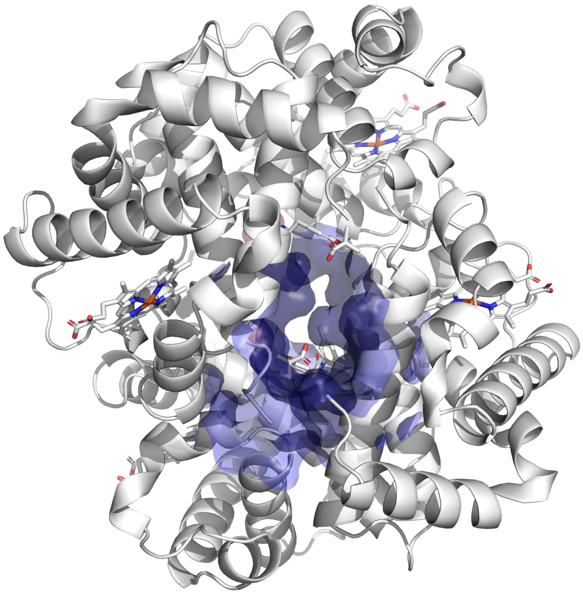

(c) DeepDock: Protein surface mesh nodes within 10 Å of any crystal ligand atom.

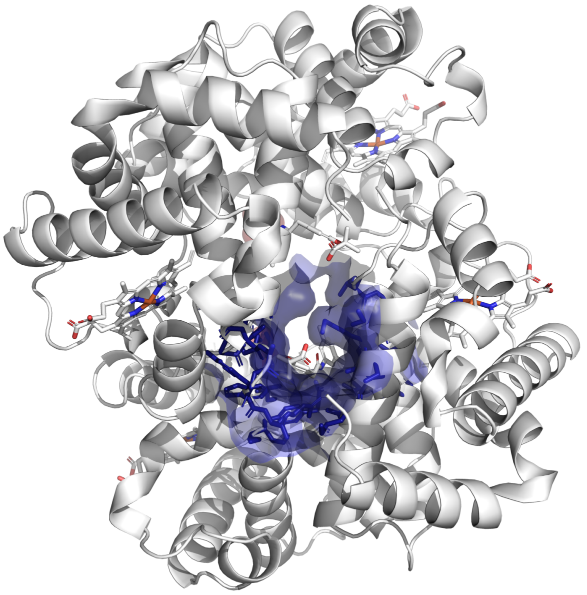

(d) Uni-Mol: Protein residues within 8 Å of any crystal ligand heavy atom.

Figure S1: Search spaces of the docking methods illustrated on PDB entry 1G9V for ligand RQ3. The search spaces for the blind docking methods DiffDock, EquiBind, and TankBind are the entire protein crystal structure. For more information refer to Table 3 in the main text.

S3 PoseBusters Benchmark set procurement

Table S1: Selection process of the PDB entries and ligands for the PoseBusters Benchmark set. The filters are based on the PDB meta data, the PDB quality reports, and the PDB structure data. The final PoseBusters Benchmark set consists of 308 unique PDB entries containing 308 unique ligands.

Selection step	Number of proteins (unique PDB IDs)	Number of ligands (unique CCD IDs)
PDB entries with a protein and 'ligand of interest' released from 1 January 2021 to 30 May 2023	10537	6635
Ligands weighing from 100 Da to 900 Da	10537	6424
Ligands with at least 3 heavy atoms	10537	6374
Ligands containing only H, C, O, N, P, S, F, Cl atoms	10537	6271
Ligands that are not covalently bound to protein	7247	4891
Structures with no unknown atoms (e.g. element X)	7218	4881
X-ray structure high resolution limit at most 2 Å	4686	3314
Ligand real space R-factor is at most 0.2	3800	2572
Ligand real space correlation coefficient is at least 0.95	1849	1054
Ligand model completeness is 100%	1820	1039
Ligand starting conformation could be generated with ETKDGv3 [3]	1733	1019
All ligand SDF files can be loaded with RDKit [4] and pass its sanitization	1706	994
PDB ligand report does not list stereochemical errors	1706	994
PDB ligand report does not list any atomic clashes	1256	844
Select single protein-ligand conformation[1]	1256	844
Intermolecular distance between the ligand(s) of interest and the protein is at least 0.2 Å	1237	834
Intermolecular distance between ligand(s) of interest and other small organic molecules is at least 0.2 Å	1237	834
Intermolecular distance between the ligand(s) of interest and ion metals in complex is at least 0.2 Å	1232	832
Blocklist for PDB entries[2]	1227	827
Blocklist for CCD entries[3]	1223	823
Randomly select PDB entries to get a set with unique ligands	809	823
Randomly select ligands to get a set with unique PDB entries	809	809
Select representative PDB entries by clustering protein sequences[4]	428	428
Remove ligands which are within 5.0 Å of any protein symmetry mate	308	308

[1] The first conformation containing the ligand of interest was chosen when multiple conformations containing the ligand were available in the PDB entry.

[2] The blocklist for the PDB entries (by PDB identifier) contains entries removed due to bad ligand conformations (7X48, 7UYC), ligands forming polymers (7WJD, 7DB4), racemeic mixtures of ligands where the stereoisomer has a different CCD identifier (6ZYU, 7W2W), and structures containing elements Te and Yb which AutoDock Vina does not support by default (7ZSQ, 8AVA).

[3] The blocklist for the ligands (by CCD identifier) contains the four entries I8P, 5A3, U71, and UEV. These four are omitted because they are highly symmetric and the substructure search yields many possible atom-atom mappings between conformations negatively affecting the RMSD calculation time.

[4] Clustering with Diamond [5] is done with an identity cutoff for the clustering of 0% and a minimum coverage of the cluster member sequences by the representative sequences of 100% and otherwise default values which includes the `BLOSUM62` substitution matrix.

S4 Data sets description

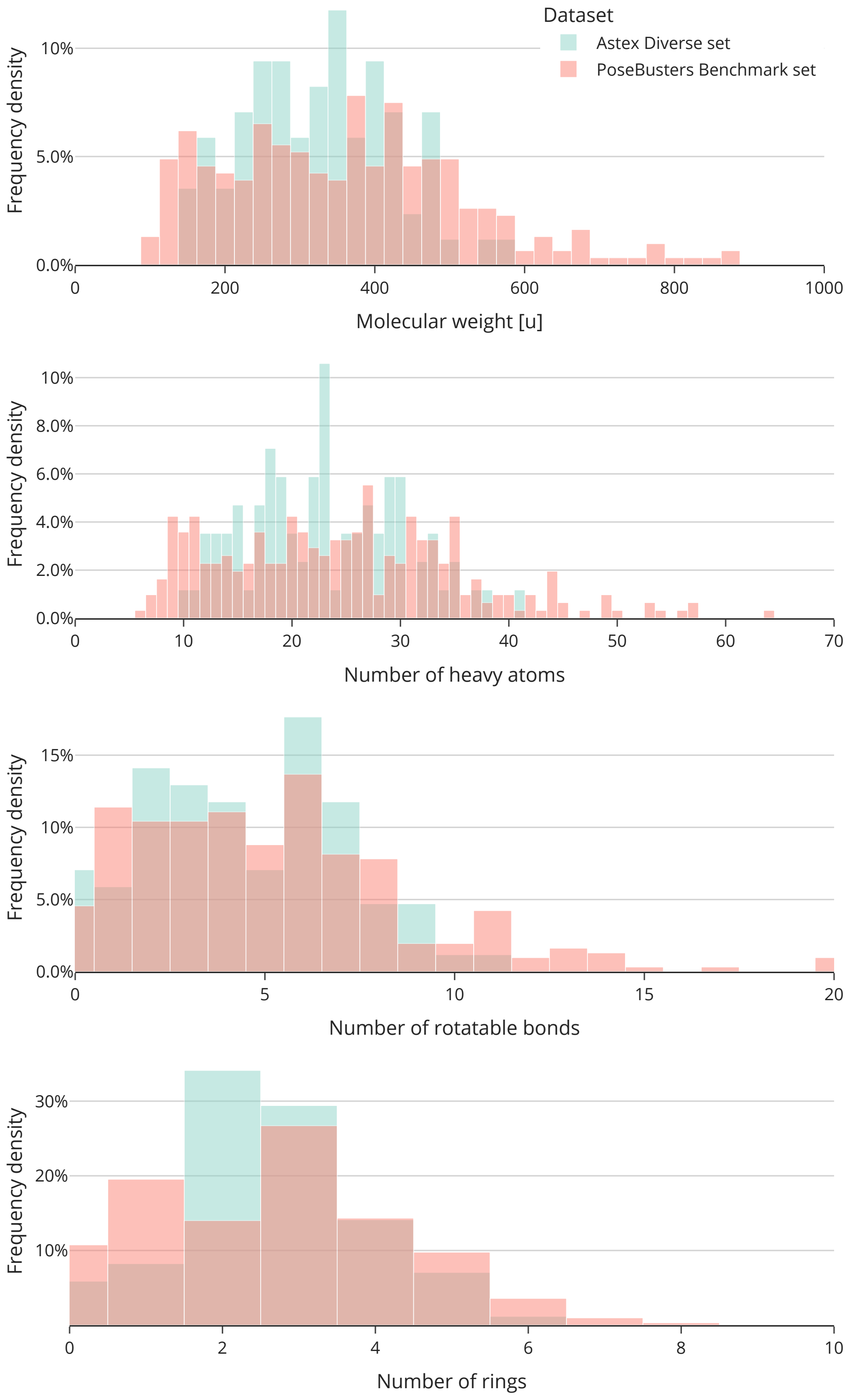

Figure S2: Comparison of the 85 ligands in the Astex Diverse set and the 308 ligands in the PoseBusters Benchmark set in terms of molecular weight, number of heavy atoms, number of rotatable bonds, and number of rings.

S5 Data sets

The following sections list the protein database[6] (PDB) codes and chemical component dictionary[7] (CCD) codes for the protein-ligand complexes and the corresponding ligands of interest for the two data sets used.

Astex Diverse set

1G9V RQ3, 1GKC NFH, 1GM8 SOX, 1GPK HUP, 1HNN SKF, 1HP0 AD3, 1HQ2 PH2, 1HVY D16, 1HWI 115, 1HWW SWA, 1IA1 TQ3, 1IG3 VIB, 1J3J CP6, 1JD0 AZM, 1JJE BYS, 1JLA TNK, 1K3U IAD, 1KE5 LS1, 1KZK JE2, 1L2S STC, 1L7F BCZ, 1LPZ CMB, 1LRH NLA, 1M2Z DEX, 1MEH MOA, 1MMV 3AR, 1MZC BNE, 1N1M A3M, 1N2J PAF, 1N2V BDI, 1N46 PFA, 1NAV IH5, 1OF1 SCT, 1OF6 DTY, 1OPK P16, 1OQ5 CEL, 1OWE 675, 1OYT FSN, 1P2Y NCT, 1P62 GEO, 1PMN 984, 1Q1G MTI, 1Q41 IXM, 1Q4G BFL, 1R1H BIR, 1R55 097, 1R58 AO5, 1R9O FLP, 1S19 MC9, 1S3V TQD, 1SG0 STL, 1SJ0 E4D, 1SQ5 PAU, 1SQN NDR, 1T40 ID5, 1T46 STI, 1T9B 1CS, 1TOW CRZ, 1TT1 KAI, 1TZ8 DES, 1U1C BAU, 1U4D DBQ, 1UML FR4, 1UNL RRC, 1UOU CMU, 1V0P PVB, 1V48 HA1, 1V4S MRK, 1VCJ IBA, 1W1P GIO, 1W2G THM, 1X8X TYR, 1XM6 5RM, 1XOQ ROF, 1XOZ CIA, 1Y6B AAX, 1YGC 905, 1YQY 915, 1YV3 BIT, 1YVF PH7, 1YWR LI9, 1Z95 198, 2BM2 PM2, 2BR1 PFP, 2BSM BSM

PoseBusters Benchmark set

5SAK ZRY, 5SB2 1K2, 5SD5 HWI, 5SIS JSM, 6M2B EZO, 6M73 FNR, 6T88 MWQ, 6TW5 9M2, 6TW7 NZB, 6VTA AKN, 6WTN RXT, 6XBO 5MC, 6XCT 478, 6XG5 TOP, 6XHT V2V, 6XM9 V55, 6YJA 2BA, 6YMS OZH, 6YQV 8K2, 6YQW 82I, 6YR2 T1C, 6YRV PJ8, 6YSP PAL, 6YT6 PKE, 6YYO Q1K, 6Z0R Q4H, 6Z14 Q4Z, 6Z1C 7EY, 6Z2C Q5E, 6Z4N Q7B, 6ZAE ACV, 6ZC3 JOR, 6ZCY QF8, 6ZK5 IMH, 6ZPB 3D1, 7A1P QW2, 7A9E R4W, 7A9H TPP, 7AFX R9K, 7AKL RK5, 7AN5 RDH, 7B2C TP7, 7B94 ANP, 7BCP GCO, 7BJJ TVW, 7BKA 4JC, 7BMI U4B, 7BNH BEZ, 7BTT F8R, 7C0U FGO, 7C3U AZG, 7C8Q DSG, 7CD9 FVR, 7CIJ G0C, 7CL8 TES, 7CNQ G8X, 7CNS PMV, 7CTM BDP, 7CUO PHB, 7D5C GV6, 7D6O MTE, 7DKT GLF, 7DQL 4CL, 7DUA HJ0, 7E4L MDN, 7EBG J0L, 7ECR SIN, 7ED2 A3P, 7ELT TYM, 7EPV FDA, 7ES1 UDP, 7F51 BA7, 7F5D EUO, 7F8T FAD, 7FB7 8NF, 7FHA ADX, 7FRX O88, 7FT9 4MB, 7JG0 GAR, 7JHQ VAJ, 7JMV 4NC, 7JXX VP7, 7JY3 VUD, 7K0V VQP, 7KB1 WBJ, 7KC5 BJZ, 7KM8 WPD, 7KQU YOF, 7KRU ATP, 7KZ9 XN7, 7L00 XCJ, 7L03 F9F, 7L5F XNG, 7L7C XQ1, 7LCU XTA, 7LEV 0JO, 7LJN GTP, 7LMO NYO, 7LOE Y84, 7LOU IFM, 7LT0 ONJ, 7LZD YHY, 7M31 TDR, 7M3H YPV, 7M6K YRJ, 7MFP Z7P, 7MGT ZD4, 7MGY ZD1, 7MMH ZJY, 7MOI HPS, 7MSR DCA, 7MWN WI5, 7MWU ZPM, 7MY1 IPE, 7MYU ZR7, 7N03 ZRP, 7N4N 0BK, 7N4W P4V, 7N6F 0I1, 7N7B T3F, 7N7H CTP, 7NF0 BYN, 7NF3 4LU, 7NFB GEN, 7NGW UAW, 7NLV UJE, 7NP6 UK8, 7NPL UKZ, 7NR8 UOE, 7NSW HC4, 7NU0 DCL, 7NUT GLP, 7NXO UU8, 7O0N CDP, 7O1T 5X8, 7ODY DGI, 7OEO V9Z, 7OFF VCB, 7OFK VCH, 7OLI 8HG, 7OMX CNA, 7OP9 06K, 7OPG 06N, 7OSO 0V1, 7OZ9 NGK, 7OZC G6S, 7P1F KFN, 7P1M 4IU, 7P2I MFU, 7P4C 5OV, 7P5T 5YG, 7PGX FMN, 7PIH 7QW, 7PJQ OWH, 7PK0 BYC, 7PL1 SFG, 7POM 7VZ, 7PRI 7TI, 7PRM 81I, 7PT3 3KK, 7PUV 84Z, 7Q25 8J9, 7Q27 8KC, 7Q2B M6H, 7Q5I I0F, 7QE4 NGA, 7QF4 RBF, 7QFM AY3, 7QGP DJ8, 7QHG T3B, 7QHL D5P, 7QPP VDX, 7QTA URI, 7R3D APR, 7R59 I5F, 7R6J 2I7, 7R7R AWJ, 7R9N F97, 7RC3 SAH, 7RH3 59O, 7RKW 5TV, 7RNI 60I, 7ROR 69X, 7ROU 66I, 7RSV 7IQ, 7RWS 4UR, 7RZL NPO, 7SCW GSP, 7SDD 4IP, 7SFO 98L, 7SIU 9ID, 7SUC COM, 7SZA DUI, 7T0D FPP, 7T1D E7K, 7T3E SLB, 7TB0 UD1, 7TBU S3P, 7TE8 P0T, 7TH4 FFO, 7THI PGA, 7TM6 GPJ, 7TOM 5AD, 7TS6 KMI, 7TSF H4B, 7TUO KL9, 7TXK LW8, 7TYP KUR, 7U0U FK5, 7U3J L6U, 7UAS MBU, 7UAW MF6, 7UJ4 OQ4, 7UJ5 DGL, 7UJF R3V, 7ULC 56B, 7UMW NAD, 7UQ3 O2U, 7USH 82V, 7UTW NAI, 7UXS OJC, 7UY4 SMI, 7UYB OK0, 7V14 ORU, 7V3N AKG, 7V3S 5I9, 7V43 C4O, 7VB8 STL, 7VBU 6I4, 7VC5 9SF, 7VKZ NOJ, 7VQ9 ISY, 7VWF K55, 7VYJ CA0, 7W05 GMP, 7W06 ITN, 7WCF ACP, 7WDT NGS, 7WJB BGC, 7WKL CAQ, 7WL4 JFU, 7WPW F15, 7WQQ 5Z6, 7WUX 6OI, 7WUY 76N, 7WY1 D0L, 7X5N 5M5, 7X9K 8OG, 7XBV APC, 7XFA D9J, 7XG5 PLP, 7XI7 4RI, 7XJN NSD, 7XPO UPG, 7XQZ FPF, 7XRL FWK, 7YZU DO7, 7Z1Q NIO, 7Z2O IAJ, 7Z7F IF3, 7ZCC OGA, 7ZDY 6MJ, 7ZF0 DHR, 7ZHP IQY, 7ZL5 IWE, 7ZOC T8E, 7ZTL BCN, 7ZU2 DHT, 7ZXV 45D, 7ZZW KKW, 8A1H DLZ, 8A2D KXY, 8AAU LH0, 8AEM LVF, 8AIE M7L, 8AP0 PRP, 8AQL PLG, 8AUH L9I, 8AY3 OE3, 8B8H OJQ, 8BOM QU6, 8BTI RFO, 8C3N ADP, 8C5M MTA, 8CNH V6U, 8CSD C5P, 8D19 GSH, 8D39 QDB, 8D5D 5DK, 8DHG T78, 8DKO TFB, 8DP2 UMA, 8DSC NCA, 8EAB VN2, 8EX2 Q2Q, 8EXL 799, 8EYE X4I, 8F4J PHO, 8F8E XJI, 8FAV 4Y5, 8FLV ZB9, 8FO5 Y4U, 8G0V YHT, 8G6P API, 8GFD ZHR, 8HFN XGC, 8HO0 3ZI, 8SLG G5A

S6 Energy minimisation example

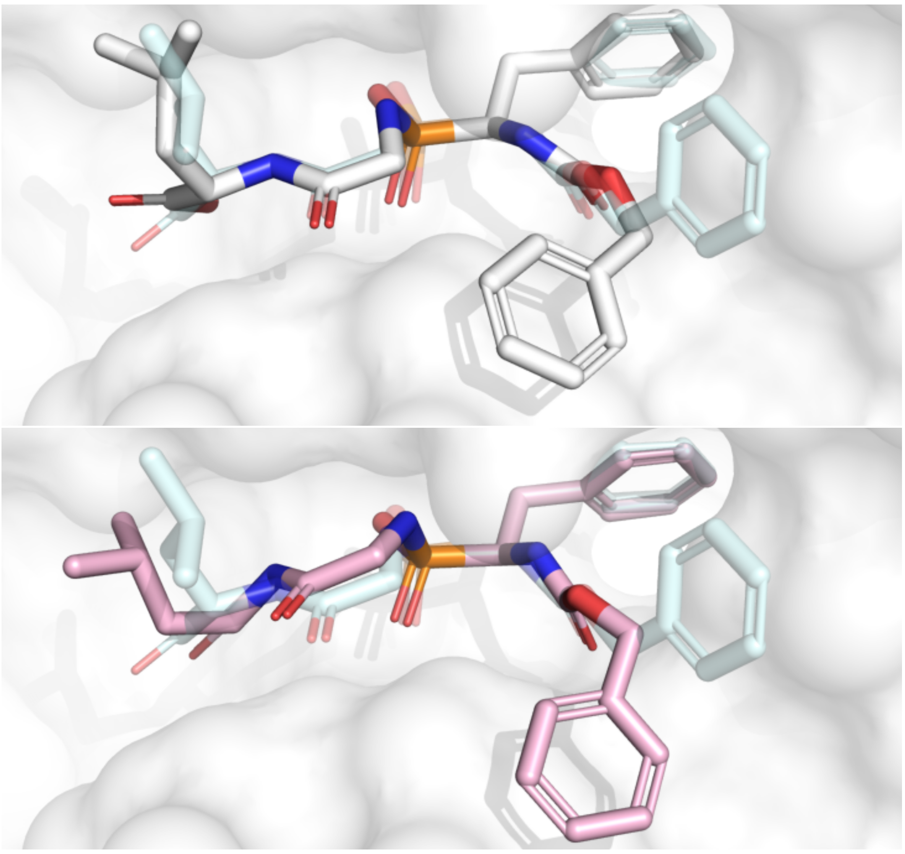

Figure S3: Example of a prediction that was ‘destroyed’ by the energy minimisation. The prediction by Autodock Vina passes all PoseBusters checks and has a RMSD of 1.9 Å and is shown in white, the optimised predicted ligand has a RMSD of 2.2 Å and is shown in pink, and the crystal ligand is shown in light blue.

S7 Cofactor analysis

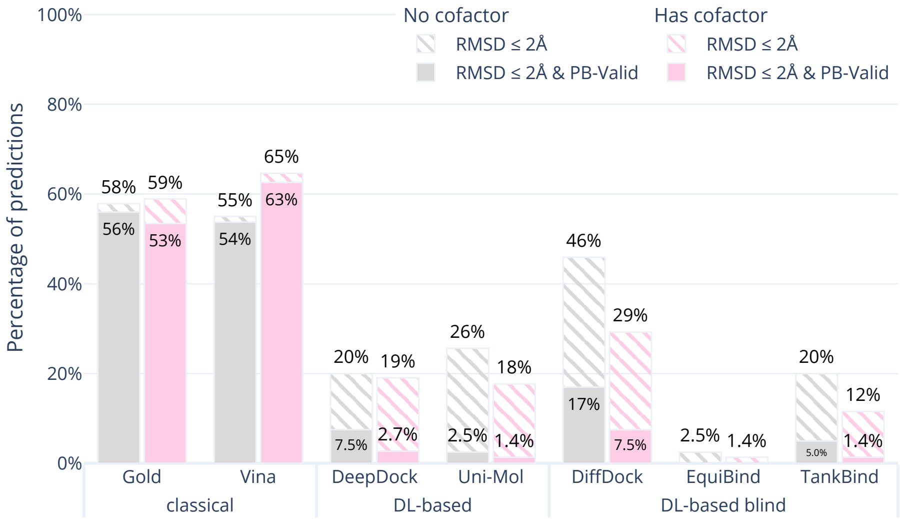

Figure S4: Comparative performance of docking methods on the PoseBusters Benchmark set stratified by the presence of cofactors. Cofactors are loosely defined as non-protein non-ligand compounds such as metal ions, iron-sulfur clusters, and organic small molecules that are within 4.0 Å of any ligand heavy atom. The striped bars show the share of predictions of each method that have an RMSD within 2 Å and the solid bars show those predictions which in addition pass all PoseBuster tests and are therefore PB-valid. The classical docking methods perform better on those systems with cofactors present while the DL-based methods perform worse on those systems.

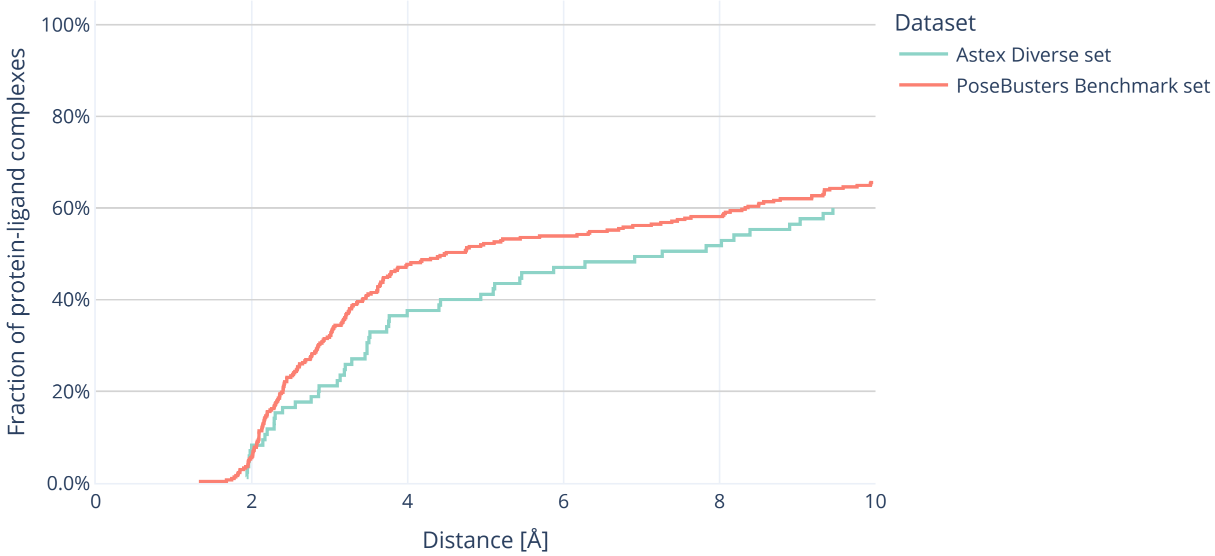

Figure S5: Fraction of protein-ligand complexes with a cofactor close to the ligand as a function of the distance threshold used. Here a cofactor is any other compound present in the crystal structure besides the ligand of interest, the protein and solvent. This includes metal ions, iron–sulfur clusters, and small organic molecules. 46% of the protein-ligand complexes in the PoseBusters Benchmark set have a cofactor within 4 Å of the ligand.

S8 Detailed results

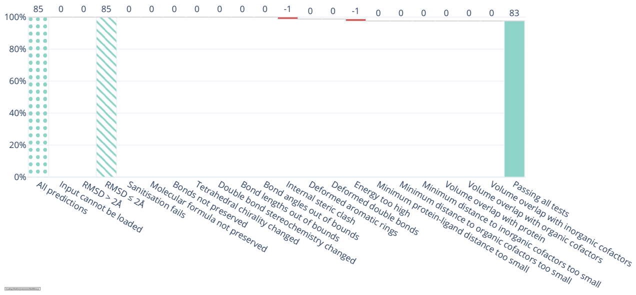

(a) Crystal Structures

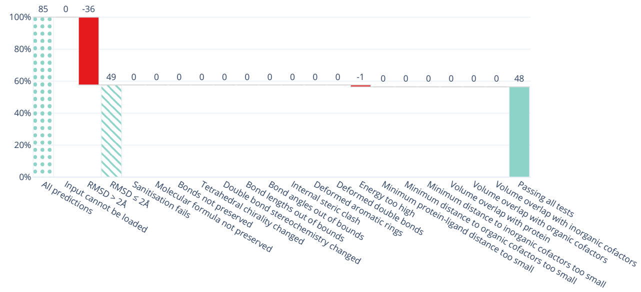

(b) AutoDock Vina

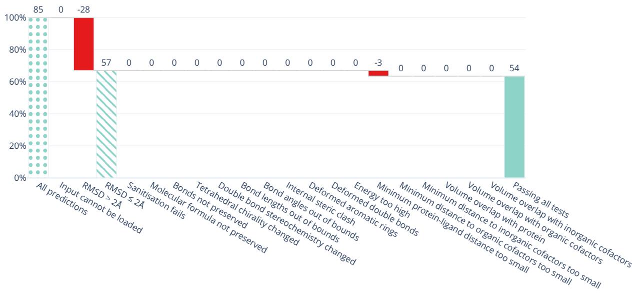

(c) CCDC Gold

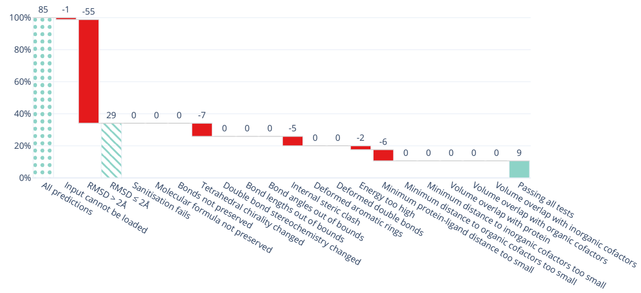

(d) DeepDock

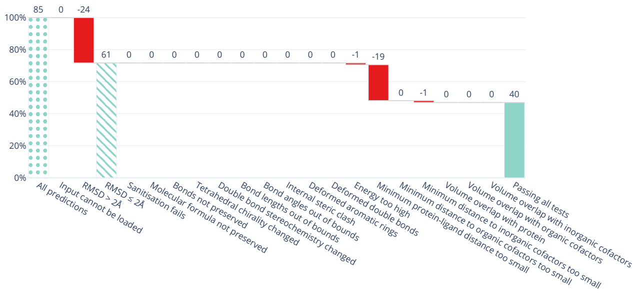

(e) DiffDock

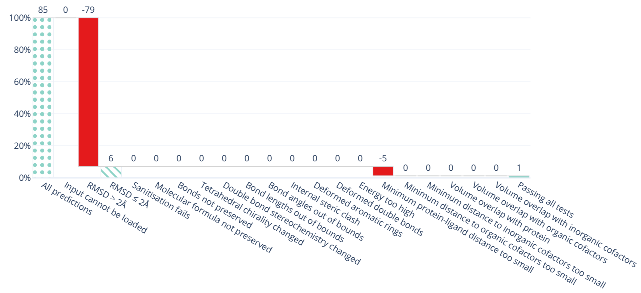

(f) EquiBind

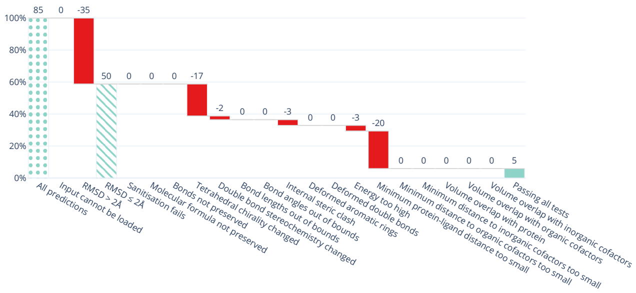

(g) TankBind

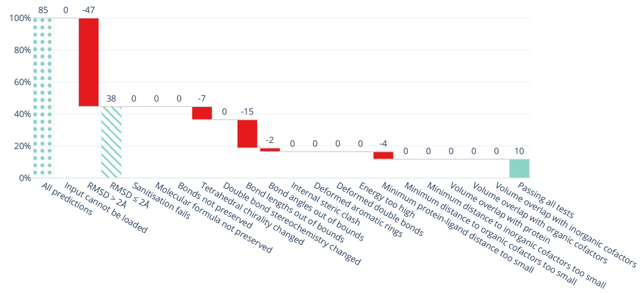

(h) Uni-Mol

Figure S6: Waterfall plots showing test results for the Astex Diverse set. The leftmost (dotted) bars show the number of complexes in the test set. The red bars show the number of predictions that fail with each additional test going from left to right. The right most (solid) bar indicates the number of predictions that pass all tests. As a reading example, panel (a) shows that out of AutoDock Vina's 85 predictions 37 are not within 2 angstrom RMSD and one additional prediction fails the energy ratio check so that overall 47 ligands have a low RMSD and pass all tests. AutoDock Vina and CCDC Gold pass the most tests.

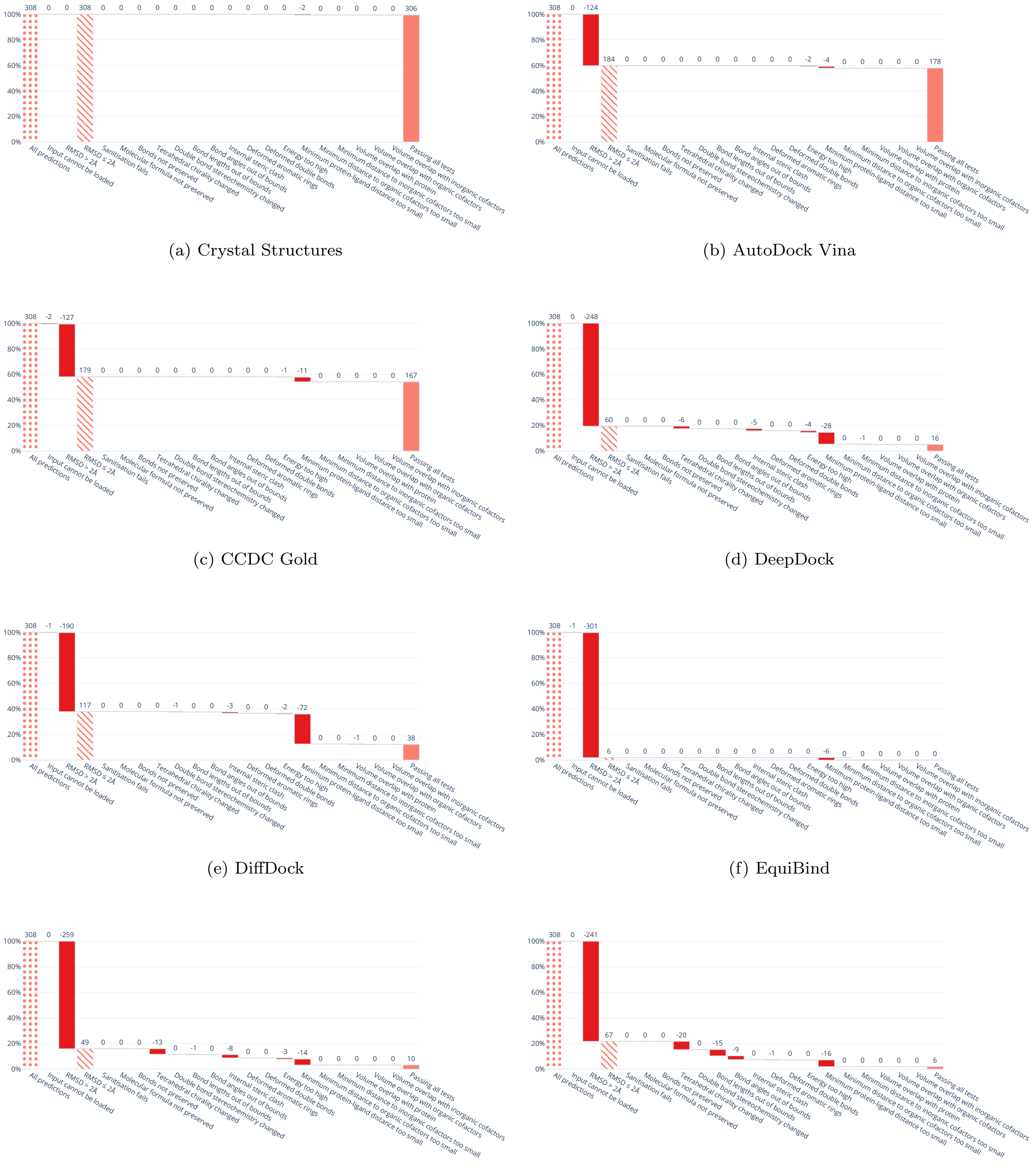

(a) Crystal Structures
(b) AutoDock Vina
(c) CCDC Gold
(d) DeepDock
(e) DiffDock
(f) EquiBind
(g) TankBind
(h) Uni-Mol

Figure S7: Waterfall plots showing test results for the PoseBusters Benchmark set. The leftmost (dotted) bar shows the number of complexes in the test set. The red bars show the number of predictions that fail with each additional test going from left to right. The right most (solid) bar indicates the number of predictions that pass all tests. Refer to the main article for a description of each test. As a reading example, panel (a) shows that out of AutoDock Vina's 308 predictions, 200 are not within 2 Å RMSD, three clash with the protein and 1 clashes with an organic cofactor leaving 224 prediction with a low RMSD passing all tests. AutoDock Vina and CCDC Gold pass the most tests.

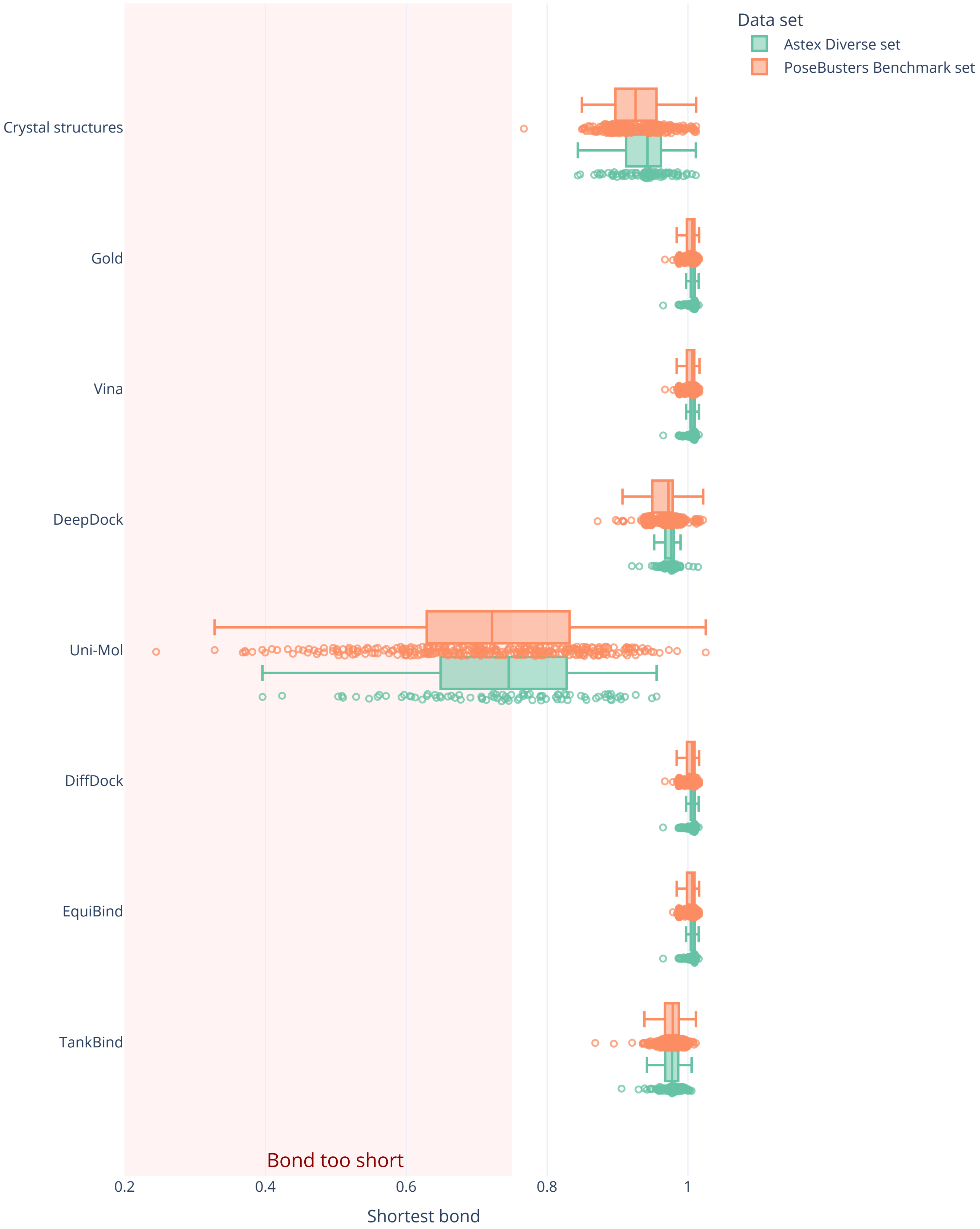

Figure S8: Distribution of shortest bond lengths. Shown are the relatively shortest bonds of each predicted ligand for each method and data set. The bond length is normalized by the lower bound for bond length obtained from Distance Geometry (DG). The lower bound correspond to one. A dot to the left of 0.75 indicates that the relatively shortest bond was more than 25% shorter than the DG lower bound. All methods except TankBind and Uni-Mol take the bond lengths from the provided ligand starting conformation. Uni-Mol and TankBind generate the bond lengths.

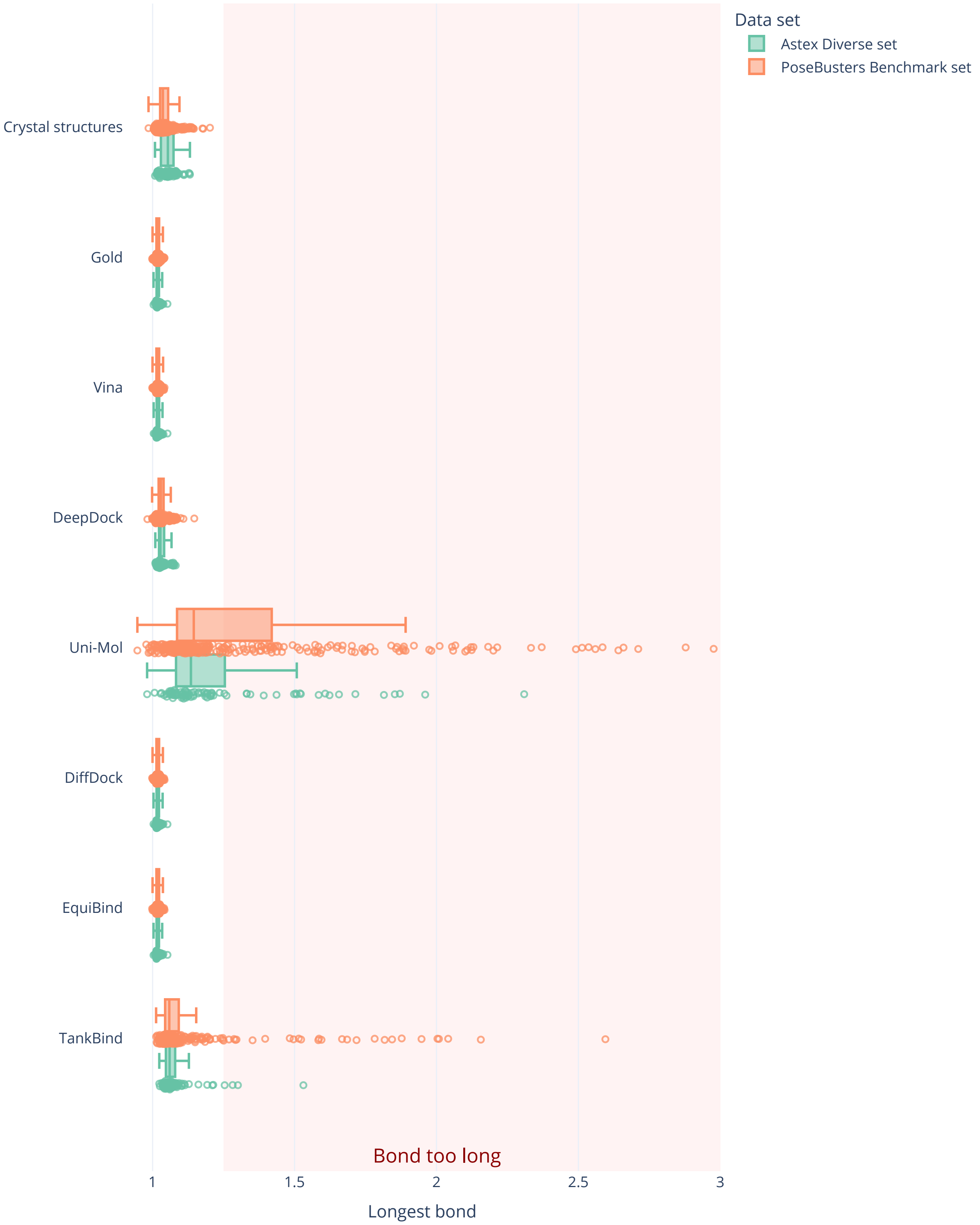

Figure S9: Distribution of longest bond lengths. Shown are the relatively longest bonds of each predicted ligand for each method and data set. The bond length is normalized by the upper bound for bond length obtained from Distance Geometry (DG). The upper bound correspond to one. A dot to the right of 1.25 indicates that the relatively shortest bond was more than 25 % longer than the DG upper bound. All methods except TankBind and Uni-Mol take the bond lengths from the provided ligand starting conformation. Uni-Mol and TankBind generate the bond lengths.

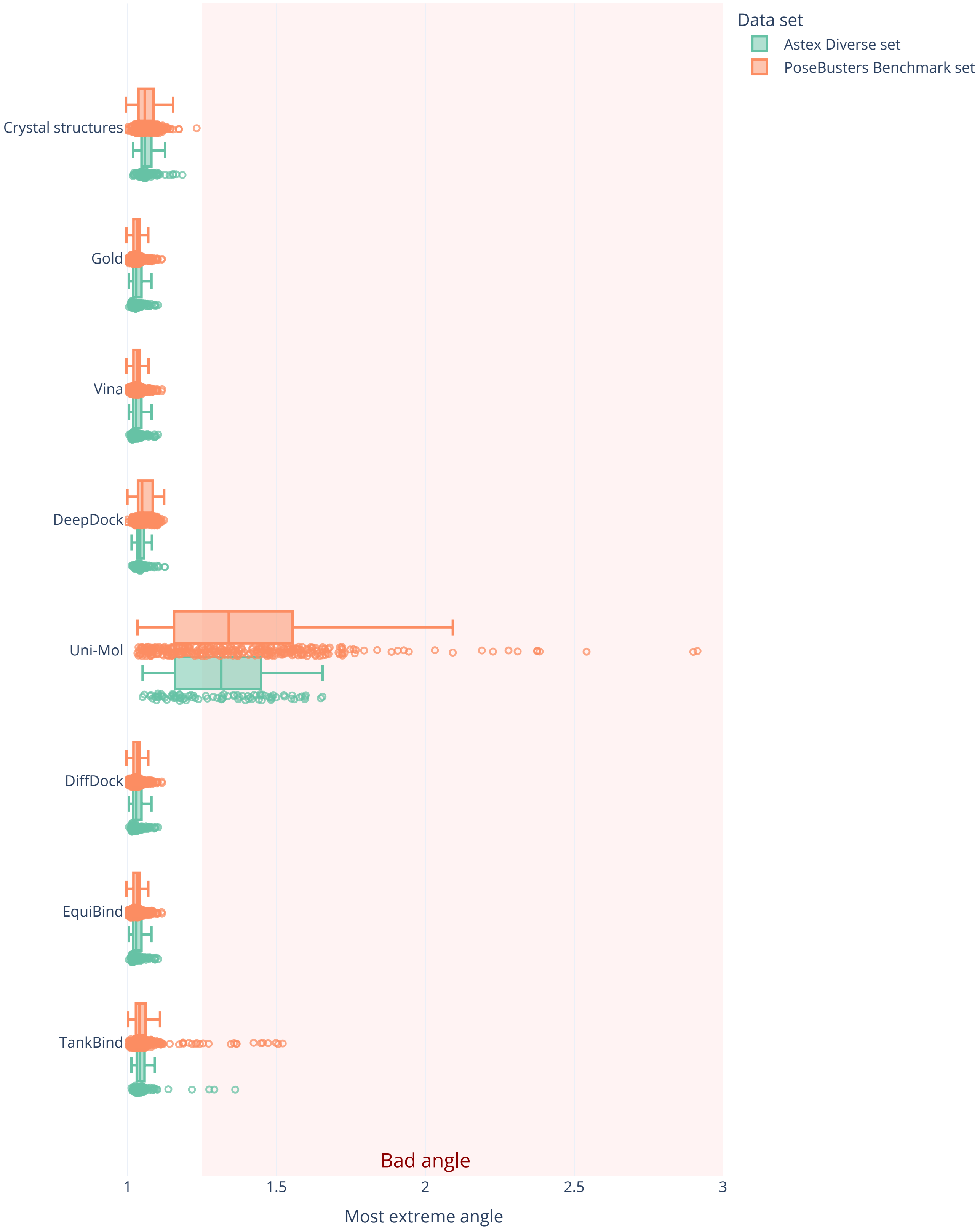

Figure S10: Distribution of relative bond angles. Shown are the most extreme angles of each predicted ligand for each method and data set. Each bond angle is normalized by the corresponding bond length bounds obtained from Distance Geometry (DG). The upper bound corresponds to one. A dot to the right of 1.25 indicates that an angle is more than 25 % larger or shorter than the DG bounds permit. All methods except TankBind and Uni-Mol take the bond angles from the provided ligand starting conformation. Uni-Mol and TankBind generate the angles.

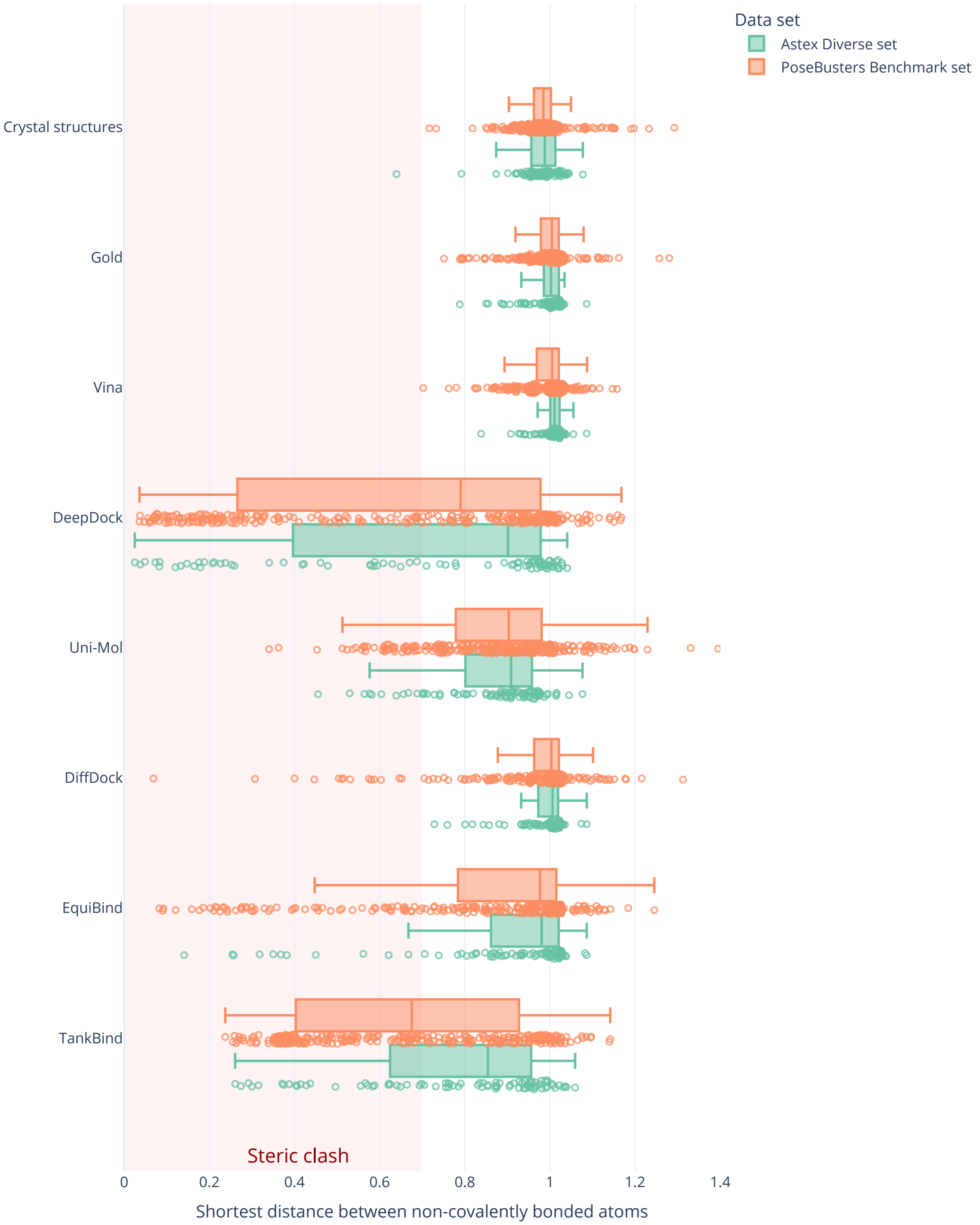

Figure S11: Distribution of distances between unbounded atoms. The distribution shows the closest pair of unbounded atoms in each predicted ligand for each method and data set. Each distance is normalized to the Distance Geometry bounds. The lower bound corresponds to one. A dot to the left of 0.7 indicates that a distance was more than 30% shorter than the lower bound and was counted as a clash.

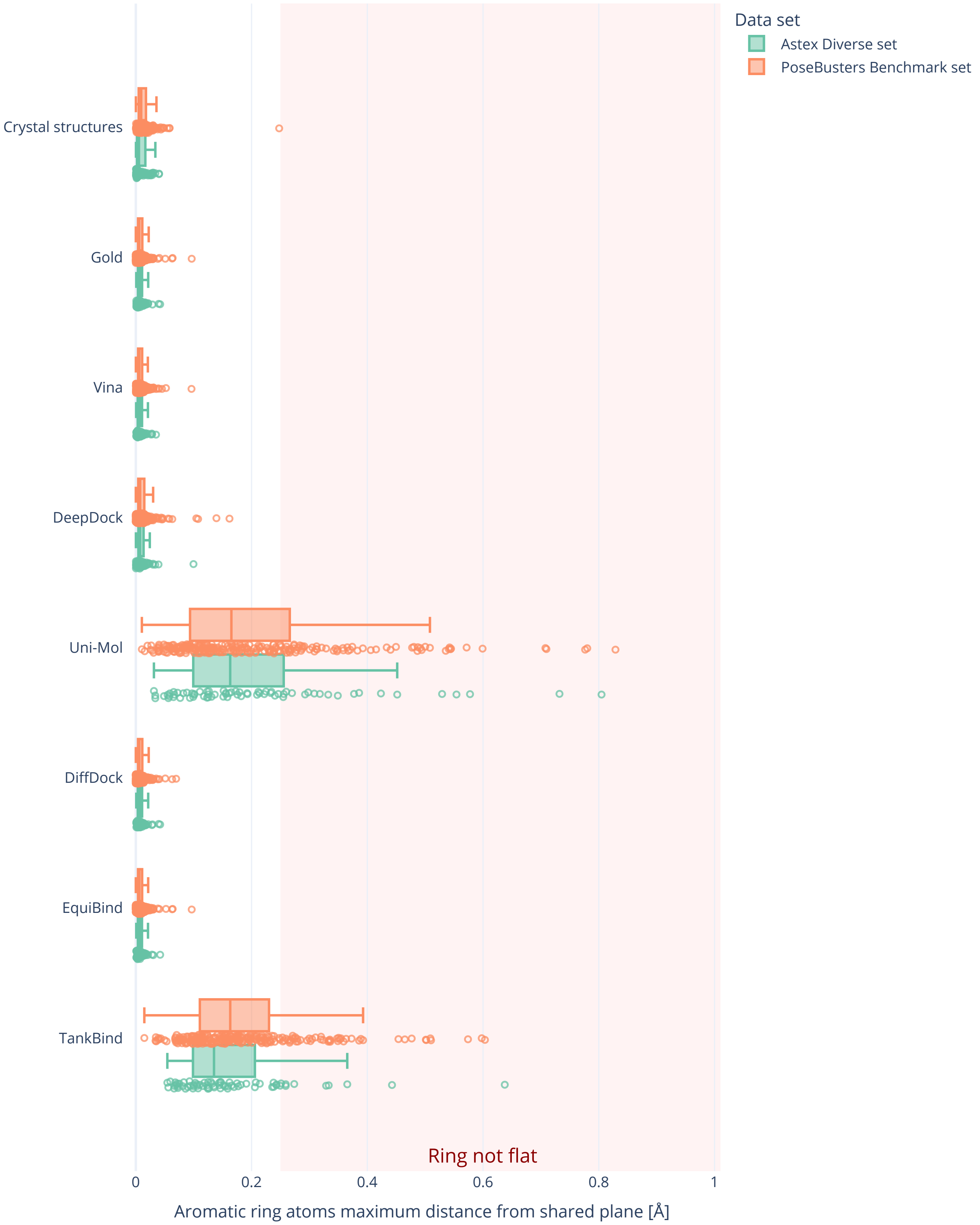

Figure S12: Distance from shared plane of atoms in 5- or 6-membered aromatic rings. The largest distance in Angstrom from the shared planed is shown for each protein ligand complex. If a ligand has no rings it is not shown. TankBind and Uni-Mol generate non-flat rings.

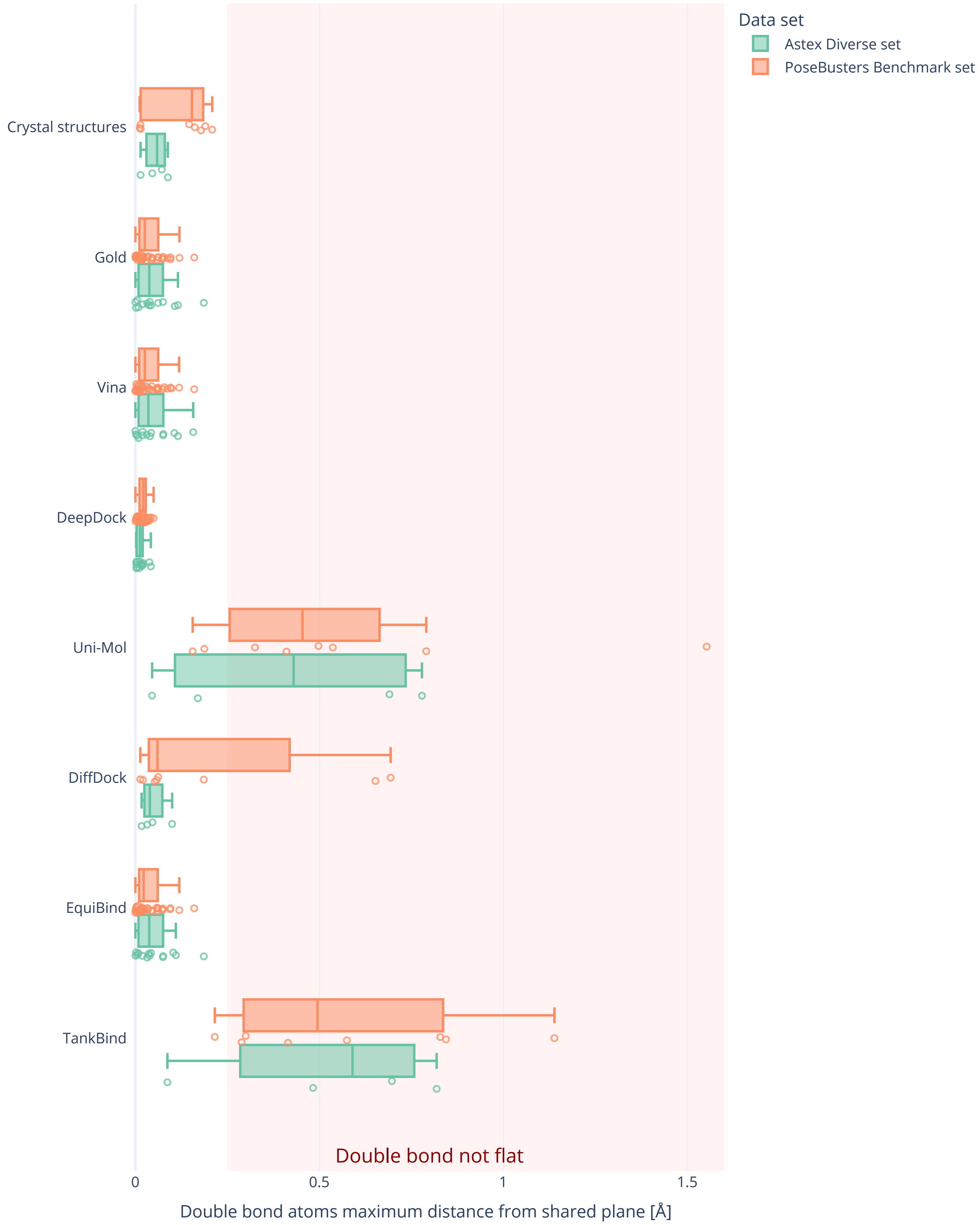

Figure S13: Distance from shared plane of atoms around aliphatic carbon-carbon double bonds. The largest distance in Angstrom from the shared planed of the two carbons and their four neighbours is shown for each protein ligand complex. If a ligand has no aliphatic carbon-carbon double bonds it is not shown. TankBind and Uni-Mol generate non-flat double bonds.

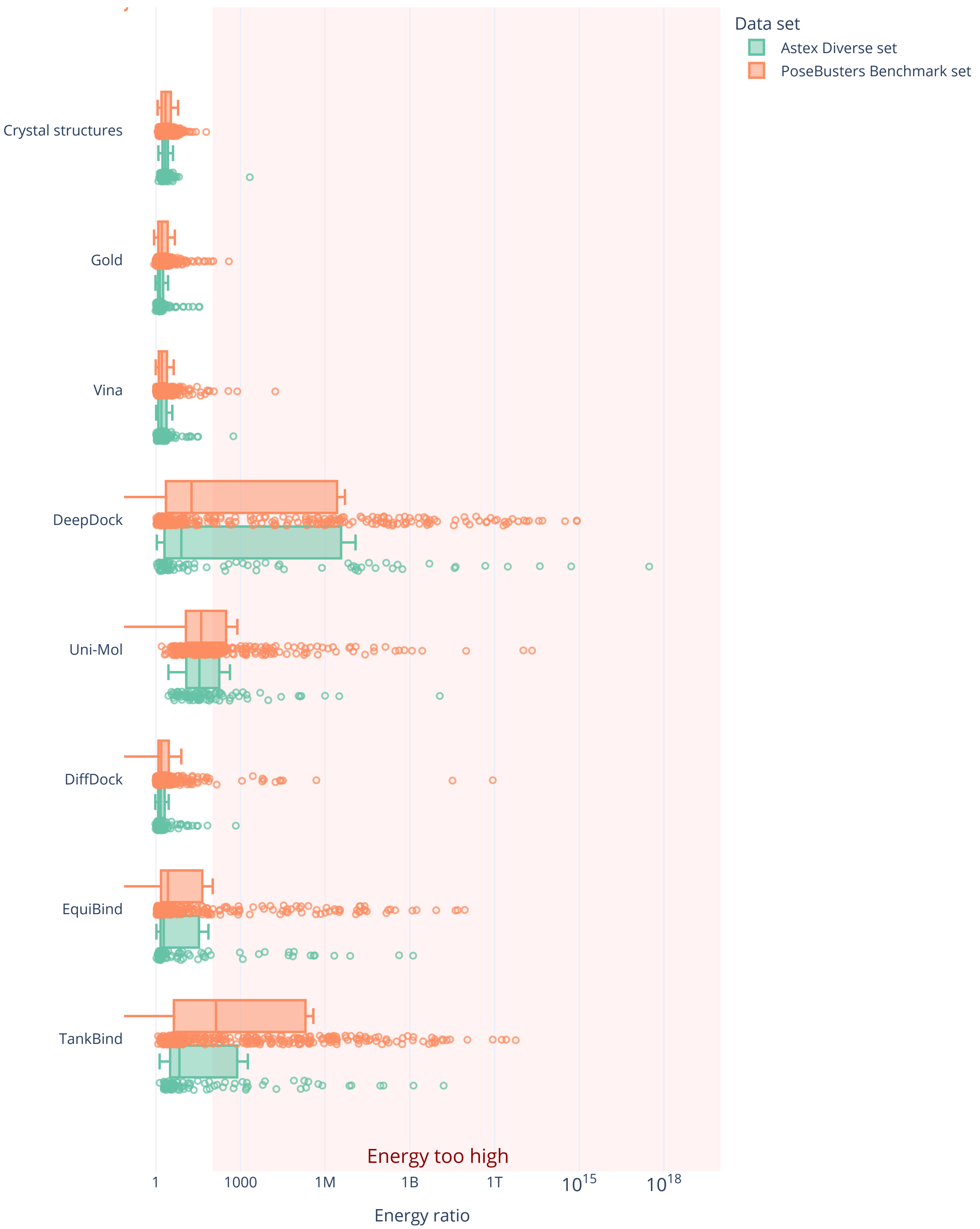

Figure S14: Energy ratio distributions. The ratio is the energy of the predicted ligand conformation over the average energy of an ensemble of 50 conformations generated with ETKDGv3. The UFF implemented in RDKit was used. The dashed red line shows the cutoff value of 100. There is only one crystal ligand in each data set with a higher energy ratio than the cutoff but all docking methods generate multiple high energy conformations above the cutoff.

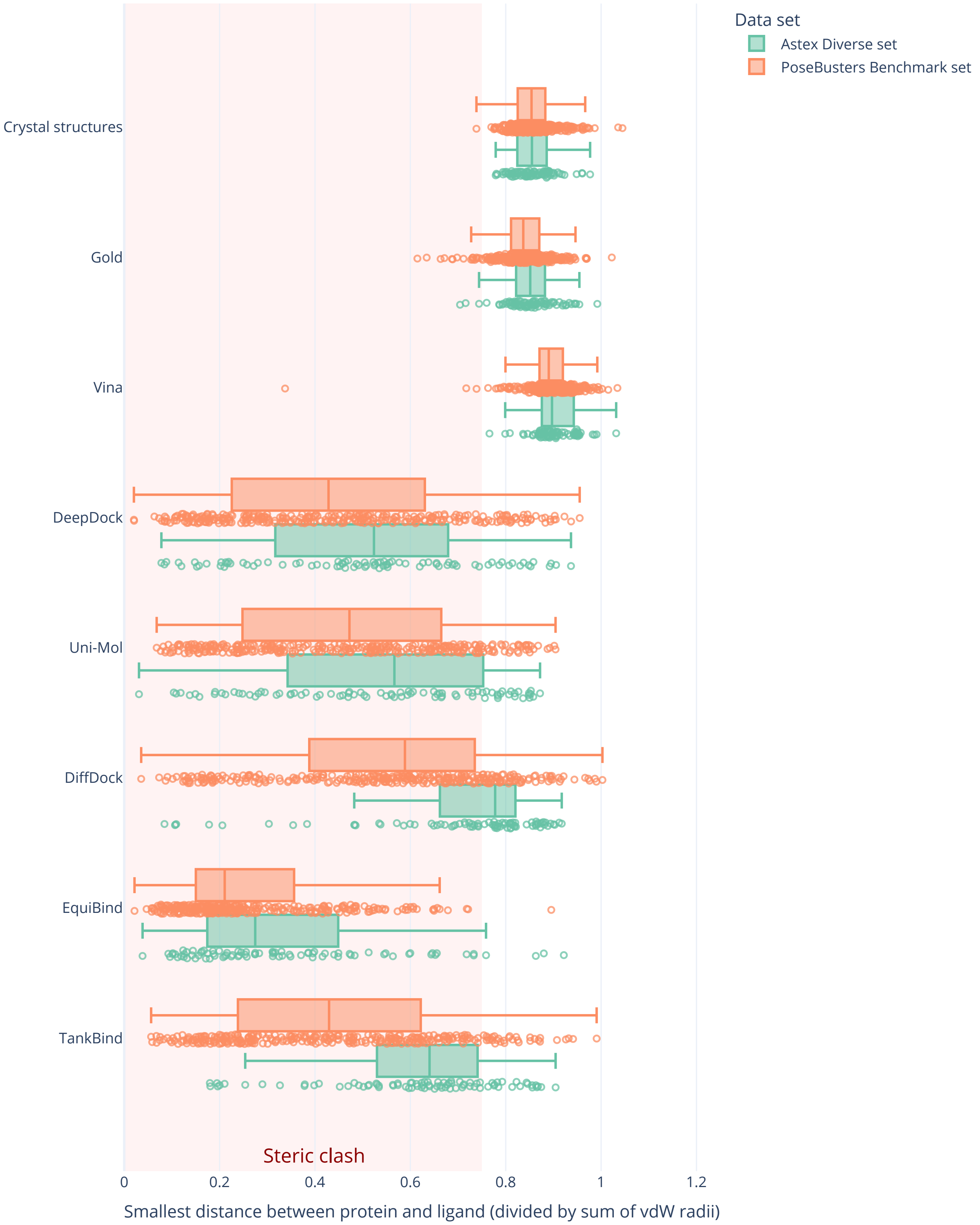

Figure S15: Minimum distances between protein and ligand. Distance is the smallest pairwise distance of heavy atoms of the ligand and protein normalized by their sum of van der Waals radii. The red area highlights the rejection zone below the cutoff of 0.75

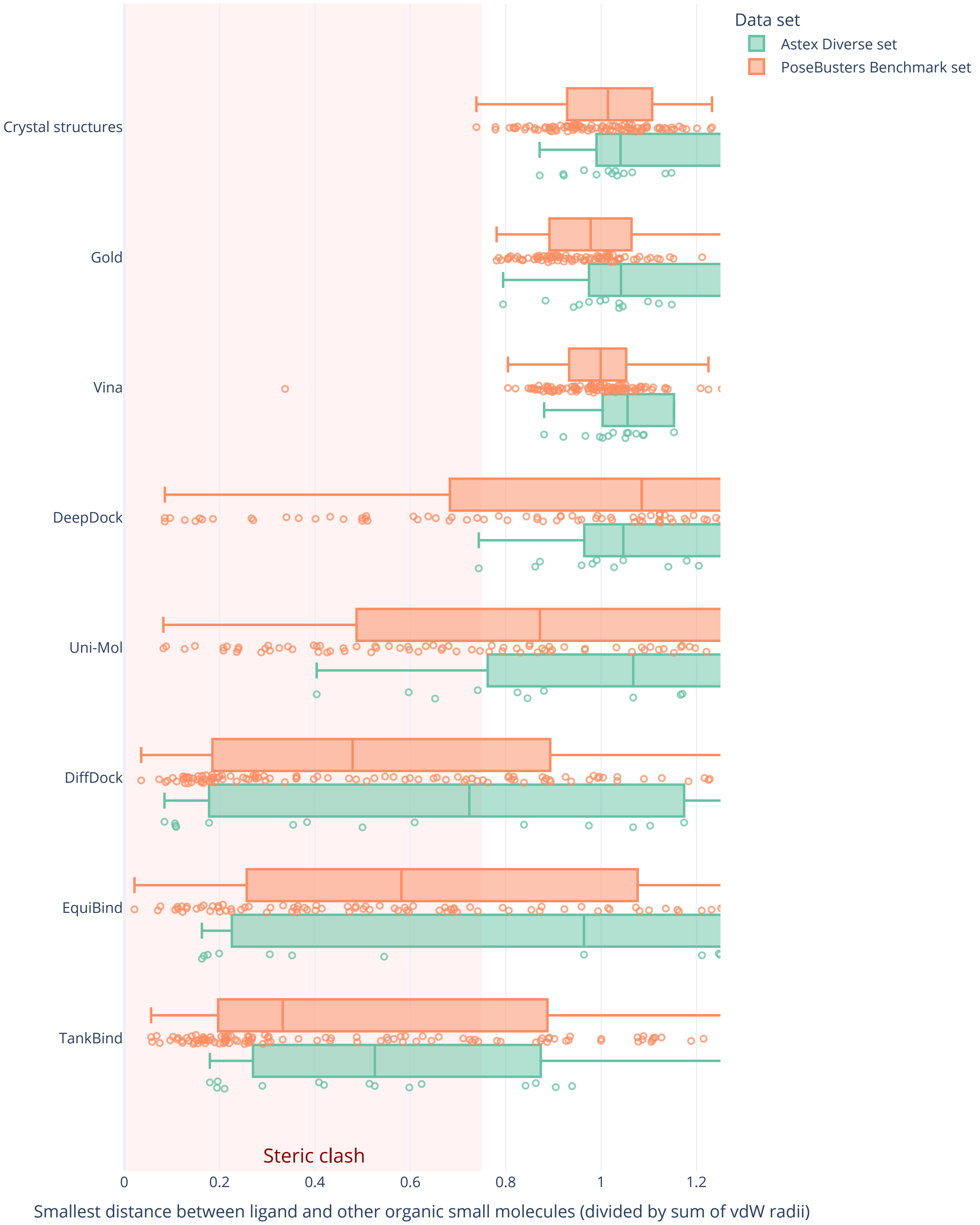

Figure S16: Minimum distances between ligand and organic molecules. Distance is the smallest pairwise distance of heavy atoms of the ligand and organic molecules normalized by their sum of van der Waals radii. The red area highlights the rejection zone below the cutoff of 0.75

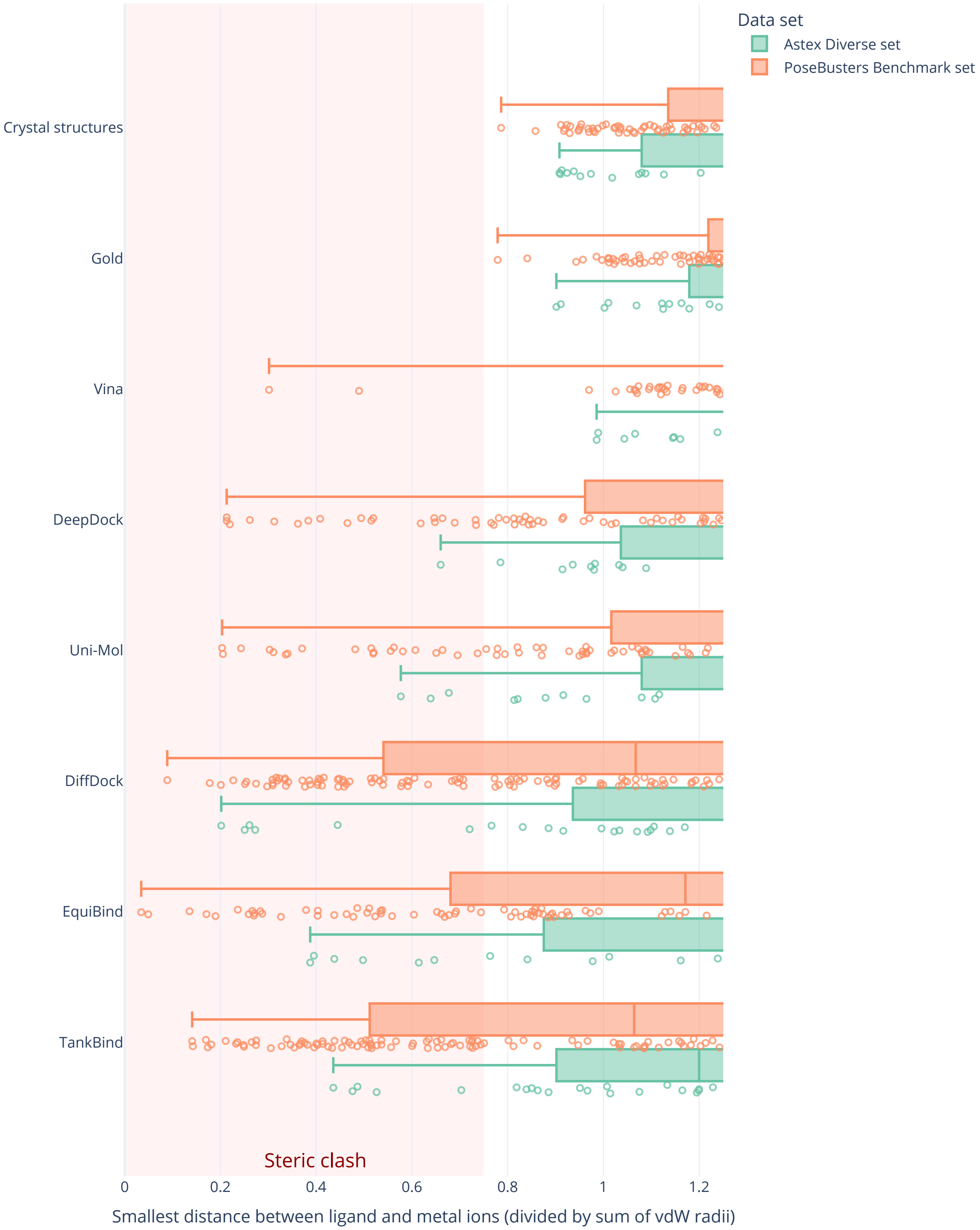

Figure S17: Minimum distances between ligand and inorganic cofactors. Distance is the smallest pairwise distance of heavy atoms of the ligand and inorganic cofactors normalized by their sum of covalent radii. The red area highlights the rejection zone below the cutoff of 0.75

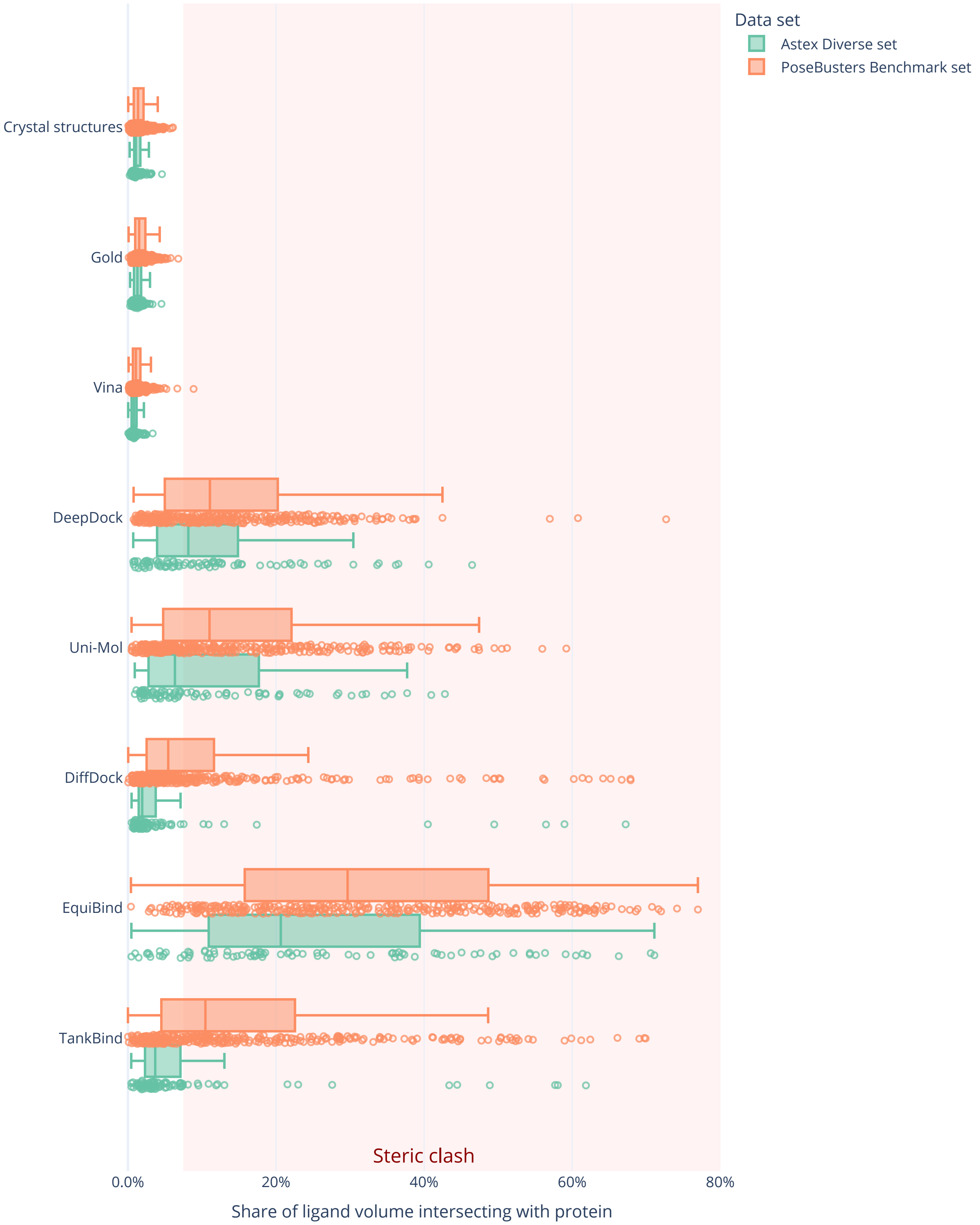

Figure S18: Volume overlap of protein and ligand. Volume overlap is the percentage of the ligand that overlaps with the protein. The volumes are the van der Waals volumes of the heavy atoms of the ligand and protein. The van der Waals radii are scaled by 0.8. The red dashed line shows the used cutoff of 5%.

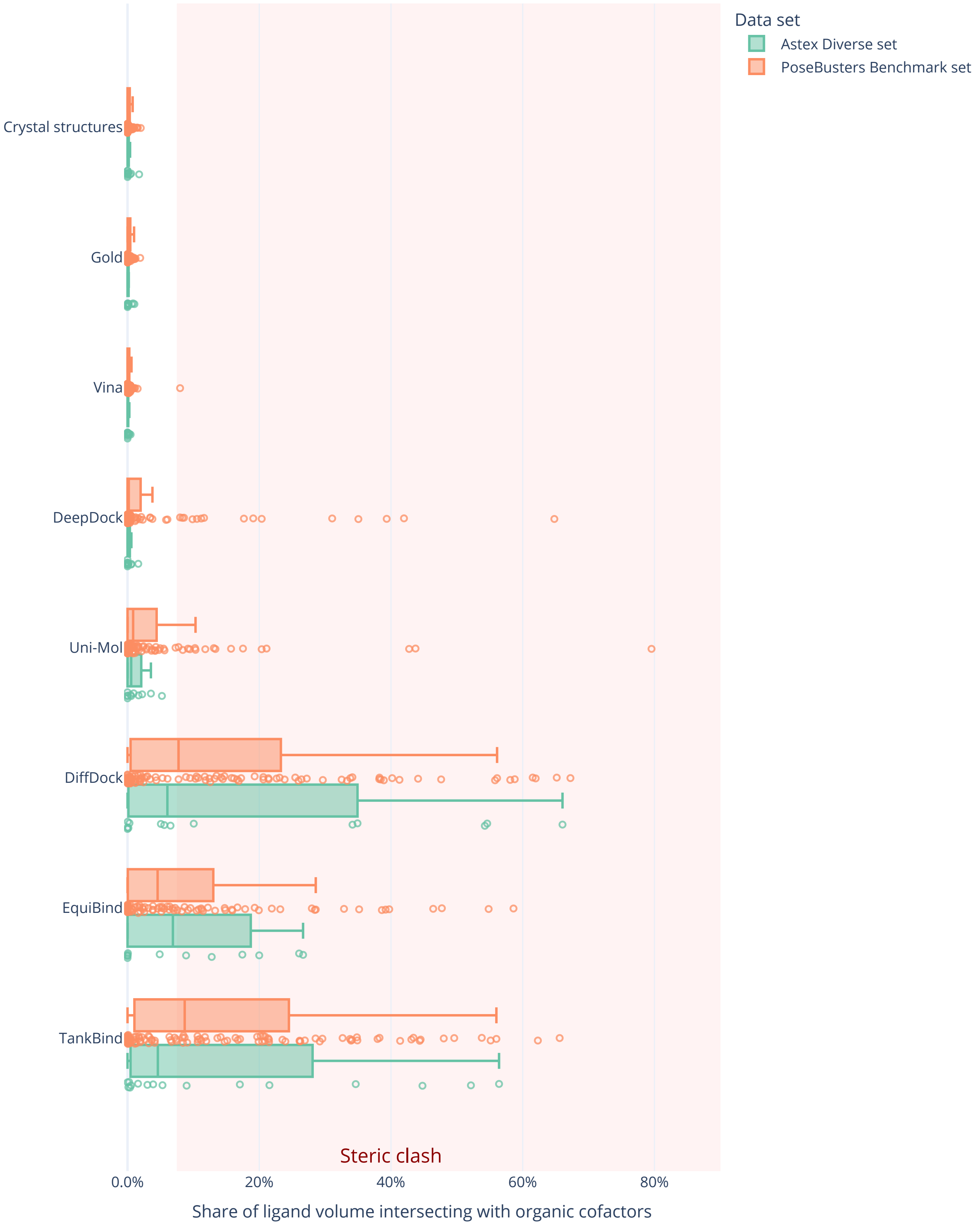

Figure S19: Volume overlap of ligand and organic molecules. Volume overlap is the percentage of the ligand that overlaps with the organic molecules. The volumes are the van der Waals volumes of the heavy atoms of the ligand and organic molecules. The van der Waals radii are scaled by 0.8. The red dashed line shows the used cutoff of 5%.

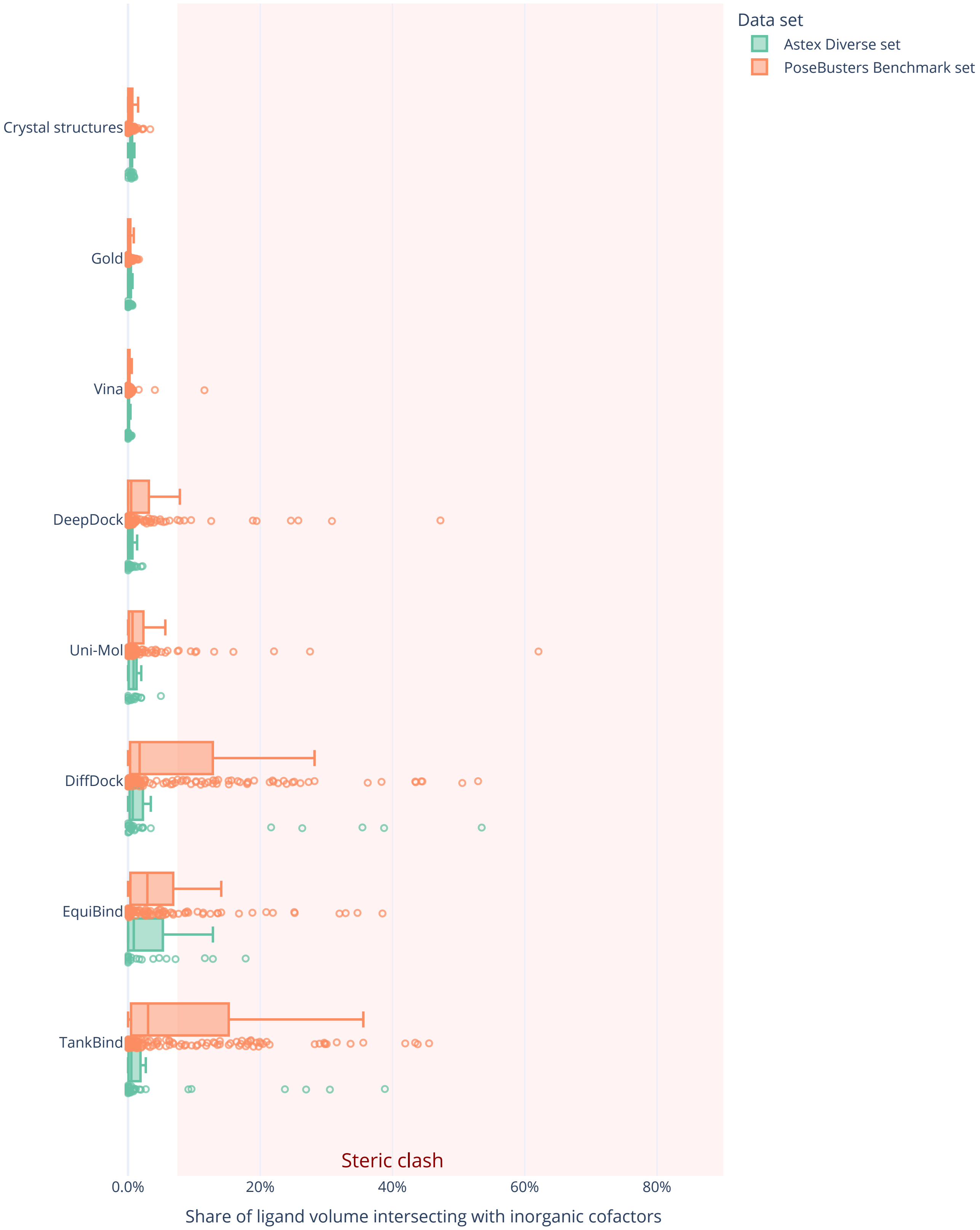

Figure S20: Volume overlap of ligand and metal ions. Volume overlap is the percentage of the ligand that overlaps with the metal ions. The volumes are the van der Waals volumes of the heavy atoms of the ligand and metal ions. The van der Waals radii are scaled by 0.8. The red zone shows the used cutoff of 20%.

S9 Alternative binding site definitions for Uni-Mol

This section explores a range of binding site definitions starting from Uni-Mol's preferred definition of all residues with an atom within 6 Å of a heavy atom of the crystal ligand. The Uni-Mol results shown here were generated using the docking procedure described in Section S1 but with different thresholds for selecting residues around the crystal ligand. The additional CCDC Gold results were generated using the procedure described in Section S1 but with a binding site selection using `BindingSiteFromLigand` with different thresholds and always selecting the entire residues.

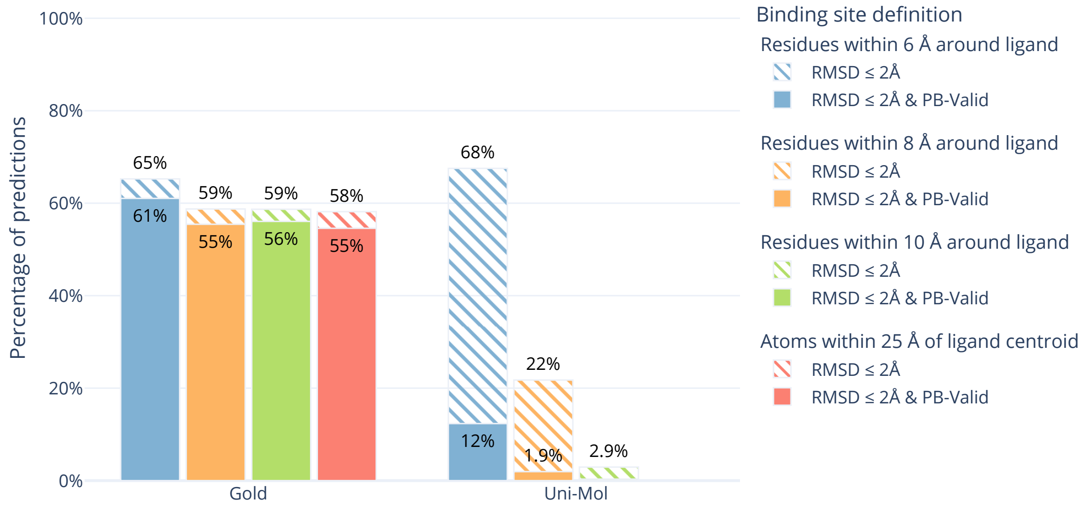

Figure S21: Performance of CCDC Gold and Uni-Mol on the PoseBusters Benchmark set on a range of binding site definitions starting from Uni-Mol's preferred definition of all residues with an atom within 6 Å of a heavy atom of the crystal ligand. The striped bars show the share of predictions of each method that have an RMSD within 2 Å and the solid bars show the subset that in addition have valid geometries and energies, i.e., pass all PoseBuster tests and are therefore 'PB-Valid'. Under the tight pocket definition of 6 Å Uni-Mol performs better than any of the blind docking methods. In the paper (Figure 1), we show the results for Gold shown here in red defining the search space as a sphere with a radius of 25 Å centred on the crystal ligand's centroid and for Uni-Mol the results in the paper are the results shown here in orange defining the binding site as all residues with an atom within 8 Å of a crystal ligand heavy atom. We selected these search spaces to make both methods more comparable to the blind docking methods.

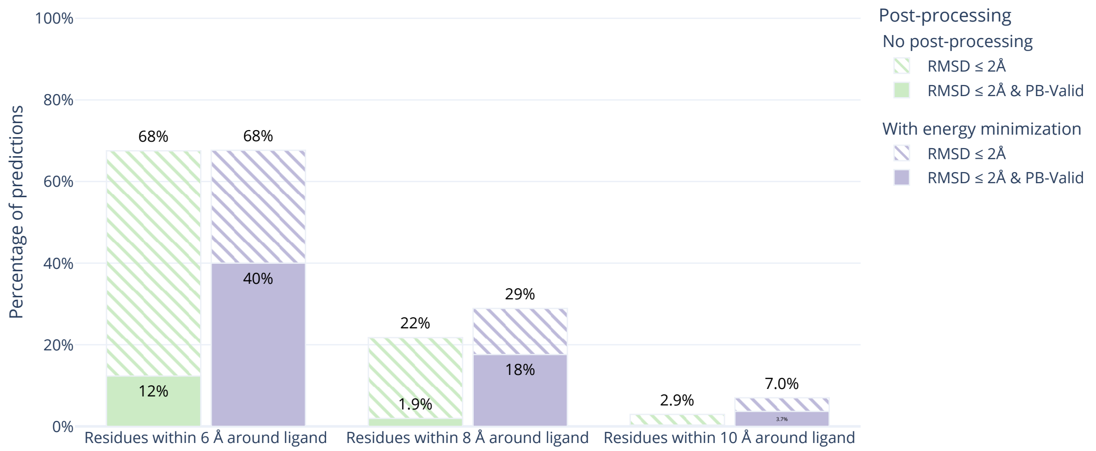

Figure S22: Performance of Uni-Mol on the PoseBusters Benchmark set for a range of binding site definitions with and without energy minimization as an additional post-processing step. The striped bars show the share of predictions of each method that have an RMSD within 2 Å of the crystal pose and the solid bars show those predictions which in addition pass all PoseBuster tests and are therefore PB-valid. Under the tight binding site definition the number of poses after energy minimisation that pass the tests increases to about the same level as DiffDock (35 %, Figure 5).

Supplementary information references

[1] S. Wang, J. Witek, G. A. Landrum and S. Riniker, *Journal of Chemical Information and Modeling*, 2020, **60**, 2044–2058.

[2] P. Tosco, N. Stiefl and G. Landrum, *Journal of Cheminformatics*, 2014, **6**, 37.

[3] S. Riniker and G. A. Landrum, *Journal of Chemical Information and Modeling*, 2015, **55**, 2562–2574.

[4] G. Landrum, P. Tosco, B. Kelley, Ric, Sriniker, Gedeck, D. Cosgrove, R. Vianello, NadineSchneider, E. Kawashima, D. N, A. Dalke, G. Jones, B. Cole, M. Swain, S. Turk, AlexanderSavelyev, A. Vaucher, M. Wójcikowski, I. Take, D. Probst, V. F. Scalfani, K. Ujihara, G. Godin, A. Pahl, F. Berenger, JLVarjo, Jasondbiggs, Strets123 and JP, *RDKit Q3 2022 Release*, Zenodo, 2023.

[5] B. Buchfink, K. Reuter and H.-G. Drost, *Nature Methods*, 2021, **18**, 366–368.

[6] H. M. Berman, *Nucleic Acids Research*, 2000, **28**, 235–242.

[7] J. D. Westbrook, C. Shao, Z. Feng, M. Zhuravleva, S. Velankar and J. Young, *Bioinformatics*, 2015, **31**, 1274–1278.
